# High-Fat Diet Remodels Plasma Membrane Rigidity to Potentiate Colonocyte Stemness in Mice

**DOI:** 10.1016/j.jcmgh.2026.101878

**Published:** 2026-09-04

**Authors:** Michael L. Salinas, Natividad R. Fuentes, Xiaoli Wang, Ellen Ruth Alexander Morris, Yang-Yi Fan, Madison E. Edwards, Xin Yan, Evelyn S. Callaway, Kerstin K. Landrock, James J. Cai, Robert S. Chapkin

**Affiliations:** 1 Program in Integrative Nutrition and Complex Diseases, Texas A&M University, College Station, Texas; 2 Department of Nutrition, Texas A&M University, College Station, Texas; 3 CPRIT Single Cell Data Science Core, Texas A&M University, College Station, Texas; 4 Interdisciplinary Faculty of Toxicology Program, Texas A&M University, College Station, Texas; 5 Department of Chemistry, Texas A&M University, College Station, Texas; 6 Department of Veterinary Integrative Biosciences, Texas A&M University, College Station, Texas; 7 Department of Electrical and Computer Engineering, Texas A&M University, College Station, Texas; 8 Regional Center of Excellence in Cancer, Texas A&M University, College Station, Texas

**Keywords:** Cholesterol, Obesity, Receptor Nanoclustering, Single-Cell Transcriptomics

## Abstract

**Background & Aims:**

Obesity and consumption of a high-fat diet are established risk factors for colorectal cancer. However, the cellular mechanisms linking dietary lipid exposure to intestinal stem cell dysregulation remain incompletely defined. We therefore investigated whether a high-fat diet perturbs plasma membrane homeostasis to potentiate colonocyte stemness in mice.

**Methods:**

Mice were placed on a chronic 12-week high-fat diet or control (low-fat diet) feeding regimen, or a short-term 1-week high-fat diet exposure. In addition, genetically obese leptin receptor-deficient (*db/db*) vs (*db/+*) wild-type mice were examined. Membrane free cholesterol and rigidity were quantified in colonic crypts and leucine-rich repeat-containing G-protein coupled receptor 5^+^ intestinal stem cells utilizing Filipin III and Di-4-ANEPPDHQ dyes, respectively. Membrane receptor nanoclustering of epidermal growth factor receptor and low-density lipoprotein receptor–related protein 6 was assessed by super-resolution microscopy. β-catenin activation, stem cell frequency, proliferation, and organoid-forming efficiency were also evaluated by immunostaining, flow cytometry, and ex vivo organoid assays.

**Results:**

Chronic high-fat diet feeding in mice increased membrane free cholesterol content and rigidity in colonic stem cells and enhanced epidermal growth factor receptor and low-density lipoprotein receptor–related protein 6 receptor nanoclustering. This alteration in plasma membrane homeostasis resulted in the overactivation of β-catenin and expansion of the intestinal stem cell niche (eg, stem cell proliferation and organoid-forming efficiency). Notably, genetically induced obesity in *db/db* mice did not recapitulate the high-fat diet membrane phenotype, highlighting a diet-specific effect.

**Conclusions:**

Collectively, these findings identify plasma membrane remodeling as a previously unrecognized mechanism linking dietary lipids to aberrant intestinal stem cell homeostasis and colorectal cancer risk.

## Graphical abstract

**Figure:**
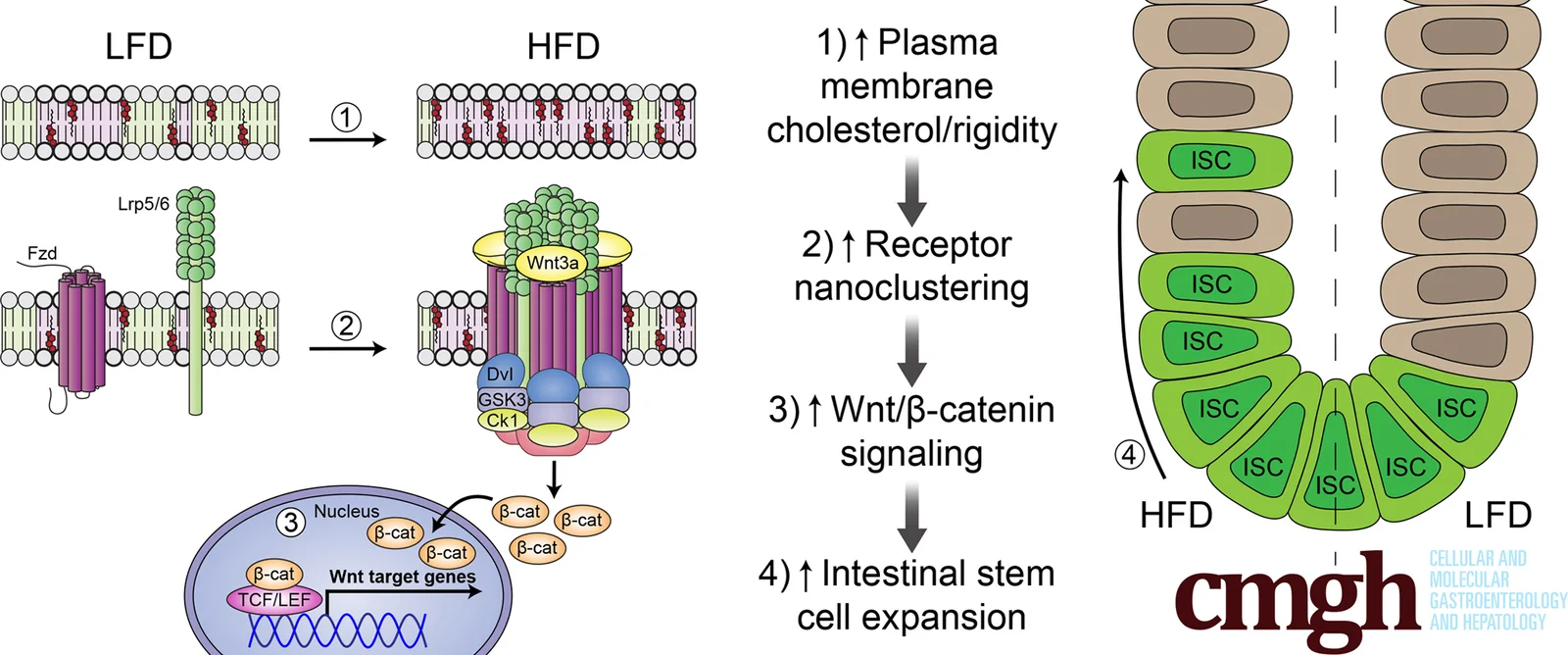

**Summary**

Chronic high-fat feeding remodels colonic epithelial plasma membrane biophysics, promoting epidermal growth factor receptor and Wnt receptor nanoclustering and β-catenin activation to expand leucine-rich repeat-containing G-protein coupled receptor 5^+^ stem cells independently of obesity. These findings suggest that membrane structure is a diet-sensitive proximal regulator of intestinal stemness.

**What You Need to Know**

**Background**

High-fat diets are associated with colorectal cancer risk, yet the upstream mechanisms linking dietary lipid exposure to intestinal stem cell expansion and tumor initiation remain incompletely defined.

**Impact**

We show that chronic high-fat feeding remodels the colonic epithelial plasma membrane, increasing cholesterol and rigidity, reorganizing receptor nanoclusters, and enhancing β-catenin signaling to expand leucine-rich repeat-containing G-protein coupled receptor 5+ stem cells in mice.

**Future Directions**

Targeting diet-induced membrane remodeling and cholesterol-dependent signaling may be a viable therapeutic strategy to maintain intestinal stem homeostasis and reduce colorectal cancer risk.

Colorectal cancer (CRC) is the second leading cause of cancer-related mortality worldwide, with rising incidence among younger individuals and populations in developing nations.^1^, ^2^, ^3^ Lifestyle factors such as the consumption of high-fat diets (HFDs) and obesity are among the strongest modifiable risk factors for CRC.^3^, ^4^, ^5^ Despite extensive epidemiologic support for this link, the cellular and molecular mechanisms by which dietary fat promotes tumor initiation remain poorly understood. Recent studies have implicated intestinal stem cells (ISCs), particularly leucine-rich repeat-containing G-protein coupled receptor 5 (Lgr5)^+^ crypt base columnar cells, as key cells-of-origin in colorectal tumorigenesis.^6^^,^^7^ Their proliferative potential, long lifespan, and susceptibility to oncogenic transformation make them a critical target for dietary modulation.^8^ Prior work has shown that chronic HFD exposure leads to expansion of the ISC compartment and enhanced tumorigenic potential,^9^, ^10^, ^11^ but the mechanisms underlying these stem cell alterations remain incompletely defined. Although many investigations have focused on how HFDs modulate metabolic signaling, systemic inflammation, or microbial dysbiosis, emerging evidence points to the plasma membrane as a direct nutrient-sensitive interface that regulates stem cell function.^12^^,^^13^ The plasma membrane’s biophysical properties, including lipid composition, cholesterol content, rigidity, and nanoscale receptor organization, profoundly influence signaling fidelity and cellular behavior.^14^, ^15^, ^16^ For example, cholesterol-rich microdomains (lipid rafts) act as dynamic signaling platforms that cluster receptors such as low-density lipoprotein receptor-related protein 6 (LRP6) and epidermal growth factor receptor (EGFR) to potentiate Wnt–β-catenin and EGFR signaling pathways, which are essential for ISC maintenance and CRC progression.^17^, ^18^, ^19^, ^20^

In the current study, we tested the hypothesis that an HFD: (1) remodels the plasma membrane of colonic epithelial cells; (2) by increasing cholesterol content and membrane rigidity; (3) thereby promoting a niche that supports stemness and proliferative capacity; (4) which subsequently promotes a stem-like epithelial state associated with increased colorectal cancer susceptibility. For this purpose, we used a combination of in vivo models, including a chronic 12-week HFD feeding regimen, a short-term 1-week HFD exposure, and genetically obese leptin receptor-deficient (*db/db*) mice to scrutinize diet- vs obesity-driven effects. In parallel, we performed ex vivo functional organoid assays, in vitro exogenous lipid assays, single-cell transcriptomics, lipidomics, and imaging-based analyses including confocal and super-resolution microscopy. Herein, we report that chronic HFD feeding increased the frequency and organoid-growing capacity of Lgr5^+^ colonic stem cells. This was associated with an increase in downstream β-catenin activation. Finally, from a mechanistic perspective, we demonstrate that HFD increased plasma membrane free cholesterol and rigidity, which promoted receptor (EGFR, LRP6) nanoclustering in Lgr5^+^ stem cells. These findings indicate a link between diet and the dysregulation of plasma membrane biochemical and biophysical properties. Surprisingly, these plasma membrane phenotypes were not observed in cells from obese *db/db* mice, despite their elevated fat mass and circulating cholesterol levels. This suggests that dietary lipids, rather than increased adiposity or hyperlipidemia, mediate the membrane remodeling responsible for altered stem cell dynamics. In support of this conclusion, we also observed similar increases in membrane rigidity in mice acutely fed an HFD (1 week), a time point prior to significant weight gain, indicating that these effects are rapid and systemic. Moreover, although treatment with saturated fatty acids (eg, palmitic acid [PA; C16:0]), failed to reproduce the membrane effects of HFD, exposure to exogenous low-density lipoprotein (LDL) cholesterol was sufficient to increase membrane cholesterol and rigidity in vitro, further implicating cholesterol as a key mediator. Collectively, our findings support a novel membrane-centric mechanism by which dietary fat rapidly alters the plasma membrane biophysical properties of colonic epithelial cells to promote ISC expansion and stem-like protumorigenic function.

## Results

### A High-Fat Diet Promotes Colonic Stem Cell Expansion and Proliferation

HFDs are strongly linked to CRC risk; however, the driving mechanisms defining the effects of chronic dietary fat exposure on colonic stem cell dynamics remain incompletely understood. Thus, we initially examined how prolonged (12 weeks) HFD (60% kcal from fat) feeding alters colonic crypt architecture and ISC behavior using Lgr5-EGFP-IRES-CreERT2 knock-in reporter mice compared with low-fat (10% kcal from fat) diet (LFD)-feeding (control) (Figure 1*A*; Table 1). Physiological characterization of mice following chronic HFD feeding (Figure 1*B–V*) confirmed expected metabolic outcomes, including increased body weight, elevated fat mass (assessed by EchoMRI), and higher circulating levels of serum total cholesterol. Although both male and female mice were exposed to the same 12-week HFD regimen, only males exhibited significant weight gain (Figure 1*B* and *C*), consistent with known sex-specific differences in susceptibility to diet-induced obesity.^21^ Therefore, all subsequent analyses of HFD and obesity-related phenotypes were restricted to male mice. Furthermore, HFD-fed mice showed no significant changes in colon length, while exhibiting a decrease in cecum and colon weight when normalized to body weight, suggesting that the observed epithelial changes were not simply attributable to increased body mass (Figure 1*N–R*).

**Figure 1:**
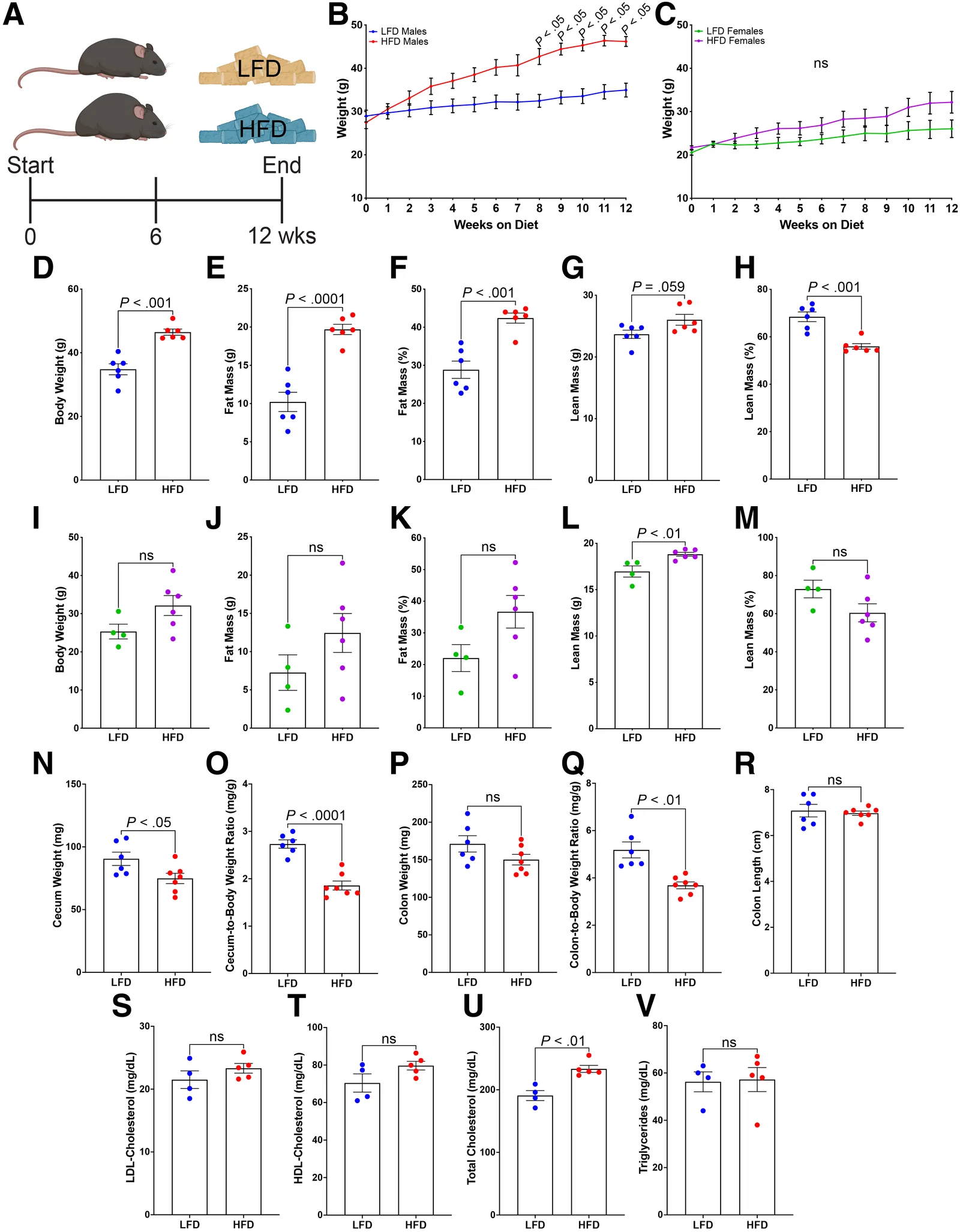
**HFD increases body weight and fat mass in male mice.** (*A*) Experimental schematic of male mice fed HFD or LFD for 12 weeks. Male (*B*) and female (*C*) mice were weighed weekly. (*D*) Male mice body weight after 12 weeks on diet. Male mice (*E* and *F*) fat mass and (*G* and *H*) lean mass were measured via echoMRI at week 12 (n = 6 mice/diet). (*I*) Female mice body weight after 12 weeks on diet. Female mice (*J* and *K*) fat mass and (*L* and *M*) lean mass were measured via echoMRI at week 12 (n = 4–6 mice/diet). Colons and ceca were collected, rinsed with DPBS, and measured (n = 6–7 male mice/diet). (*N* and *O*) Cecum weight, (*P* and *Q*) colon weight, (*R*) colon length. Blood serum cholesterol and lipid levels were quantified (n = 4–5 male mice/diet). Quantification of blood serum (*S*) LDL cholesterol, (*T*) HDL cholesterol, (*U*) total cholesterol, and (*V*) triglycerides (n = 4–5 male mice/diet). Data are presented as mean ± standard error of the mean. Statistical significance between treatments was assessed by (*B* and *C*) Holm–Sidak multiple comparisons *t* test or (*D–V*) an unpaired 2-tailed Student *t* test. ns, not significant, *P* > .05. Components of panel (*A*) were created with BioRender.com.

**Table 1:** Diet Composition

Ingredients	LFD		HFD	
	grams	kcal	grams	kcal
Casein	200	800	200	800
L-cystine	3	12	3	12
Corn starch	315	1260	0	0
Maltodextrin 10	35	140	125	500
Sucrose	350	1400	68.8	275
Cellulose	50	0	50	0
Soybean oil	25	225	25	225
Lard	20	180	245	2205
Mineral mix S10026	10	0	10	0
DiCalcium phosphate	13	0	13	0
Calcium carbonate	5.5	0	5.5	0
Potassium citrate	16.5	0	16.5	0
Vitamin mix V10001	10	40	10	40
Choline bitartrate	2	0	2	0
FD&C yellow dye #5	0.05	0	0	0
FD&C blue dye #1	0	0	0.05	0
Total	1055.05	4057	773.85	4057
Cholesterol content, *μg/kcal*	13.41		53.34	

NOTE. Diet ingredients for LFD (10% kcal from fat; Research Diets D12450B) and HFD (60% kcal from fat; Research Diets D12492).

HFD, high-fat diet; LFD, low-fat diet.

Histologic analysis of colonic tissue from mice fed an HFD for 12 weeks revealed a significant increase in colonic crypt length compared with LFD-fed mice (Figure 2*A* and *B*), similar to previous findings that HFD promotes crypt hyperplasia in the small intestine.^9^ Immunofluorescence staining and quantification of Lgr5–green fluorescent protein (GFP) expression showed a marked increase in the number and percentage of Lgr5^+^ cells per crypt in HFD-fed mice (Figure 2*C* and *D*), suggesting an expanded ISC compartment. These data were corroborated by flow cytometry, which showed a higher proportion of Lgr5-GFP^+^ cells per colon in the HFD group (Figure 2*E*), consistent with previous findings demonstrating that an HFD increases the frequency of ISCs in the small intestine and colon.^9^ To evaluate whether these stem cells exhibited enhanced proliferative activity, we quantified the 5-ethynyl-2′-deoxyuridine (EdU)^+^ Lgr5-GFP^+^ double-positive cells per total number of EdU^+^ cells. Consistent with a previous report showing that an HFD increases the number of proliferative cells per crypt in the distal colon,^9^ HFD-fed mice displayed a higher percentage of proliferating ISCs compared with LFD-fed controls (Figure 2*F* and *G*), suggesting that the Lgr5^+^ compartment is not only expanded but also more mitotically active. Functionally, this was associated with a trend toward increased organoid-forming efficiency and a significant increase in organoid size using Lgr5-GFP^hi^ cells isolated from HFD-fed mice (Figure 2*H–J*). Notably, these effects were not observed in the Lgr5-GFP^low^ (Figure 2*K* and *L*) population, indicating that the enhanced regenerative and proliferative capacity may be more specific to ISCs. Similar findings indicate the enhanced organoid-forming capacity of colonic crypts isolated from HFD-fed mice.^9^ Together, these results indicate that chronic HFD feeding increases the frequency, proliferative capacity, and regenerative potential of colonic Lgr5^+^ stem cells, establishing a potential cellular mechanism by which dietary fat primes the colon for tumorigenesis.

**Figure 2:**
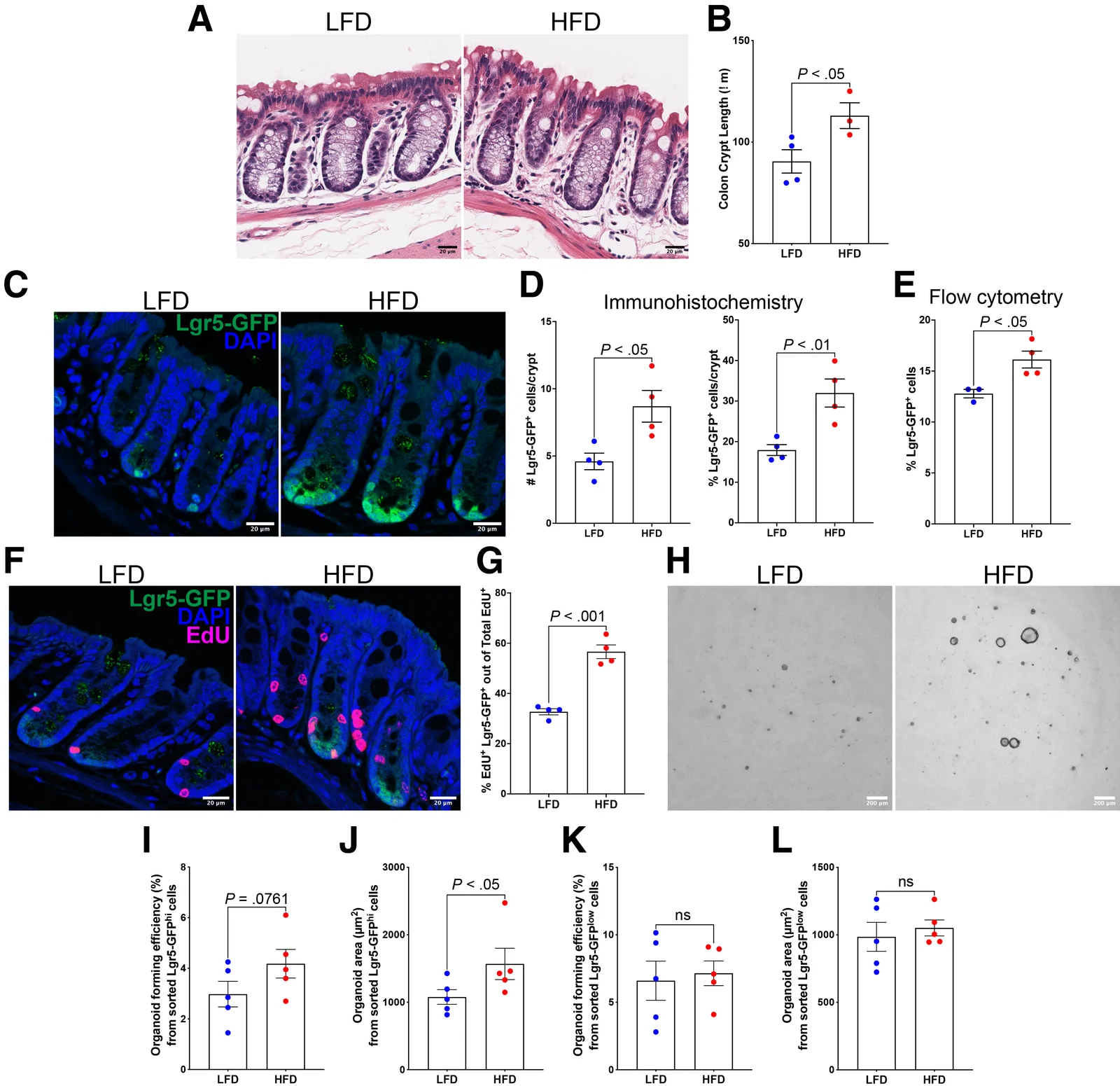
**HFD promotes colonic stem cell expansion and proliferation.** (*A*) Representative images of colon sections stained with hematoxylin and eosin (H&E) from LFD- and HFD-fed mice and (*B*) quantification of colonic crypt length (*n*=3-4 mice/diet). (C) Representative immunofluorescence images of colon sections stained with 4′,6-diamidino-2-phenylindole (DAPI) and Lgr5-GFP from LFD- and HFD-fed mice and (*D*) number and percentage of Lgr5-GFP^+^ cells per colonic crypt (n = 4 mice/diet). (*E*) Flow cytometric quantification of the percentage of colonic Lgr5-GFP^+^ cells (n = 3–4 mice/diet). (*F*) Representative immunofluorescence images of colon sections stained with DAPI, Lgr5-GFP, and EdU from LFD- and HFD-fed mice. (*G*) Quantification of the percentage of Lgr5 and EdU double positive cells relative to total EdU positive cells (n = 4 mice/diet). Sorted Lgr5-GFP^hi^ and Lgr5-GFP^low^ cells were plated in Matrigel (n = 5 mice/diet, 3 wells of organoids/mouse) and (*H*) representative images of Lgr5-GFP^hi^ colonic organoids in Matrigel from LFD- and HFD-fed mice. (*I*) Quantification of Lgr5-GFP^hi^ organoid-forming efficiency and (*J*) Lgr5-GFP^hi^ organoid area. (*K*) Quantification of Lgr5-GFP^low^ organoid-forming efficiency and (*L*) Lgr5-GFP^low^ organoid area. Data are presented as mean ± standard error of the mean. Statistical significance between treatments was assessed by an unpaired 1-tailed (*I–L*) or 2-tailed (*B, D, E* and *G*) Student *t* test. ns, not significant, *P* > .05. Scale bar (*A, C* and *F*), 20 μm, (*H*), 200 μm.

### A High-Fat Diet Elevates Plasma Membrane Cholesterol and Rigidity in Colonocytes

Plasma membrane biophysical properties, such as rigidity, cholesterol content, and organization, are critical regulators of membrane receptor organization and downstream signaling, particularly in stem cell compartments and cancer.^13^^,^^15^, ^16^, ^17^ To determine whether chronic HFD exposure alters membrane biophysics in the colon, we used Di-4-ANEPPDHQ (Di4), a polarity-sensitive dye, to assess membrane rigidity. Confocal imaging of colonic crypts revealed significantly higher generalized polarization (GP) values in HFD-fed mice compared with controls, indicating increased membrane rigidity (Figure 3*A* and *B*). To assess whether these changes extended to colonic stem and progenitor cells, we performed multispectral imaging flow cytometry (via FlowSight) on sorted Lgr5-GFP^+^ cells from HFD-fed mice that exhibited an increase plasma membrane rigidity in Lgr5^+^ cells (Figure 3*C* and *D*). This effect was mirrored and potentially driven by an increase in free cholesterol content, measured using Filipin III staining in both colonic crypts (Figure 3*E* and *F*) and in Lgr5-GFP^+^ cells (Figure 3*G* and *H*). Importantly, this approach utilized plasma membrane masking on FlowSight images to specifically measure changes at the cell surface, confirming that the observed rigidity and cholesterol enrichment were localized to the plasma membrane. To provide an orthogonal biochemical assessment of cholesterol abundance, lipids were extracted from isolated colonic crypts and analyzed using the Amplex Red Cholesterol Assay. Free (unesterified) and total cholesterol levels were significantly increased in HFD-fed mice relative to LFD controls when normalized to lipid phosphate content, whereas cholesteryl ester levels were unchanged (Figure 3*I–K*). These findings corroborate the increase in free cholesterol observed by Filipin III staining and suggest that chronic HFD feeding expands both the free and total cholesterol pools within colonic epithelial cells.

**Figure 3:**
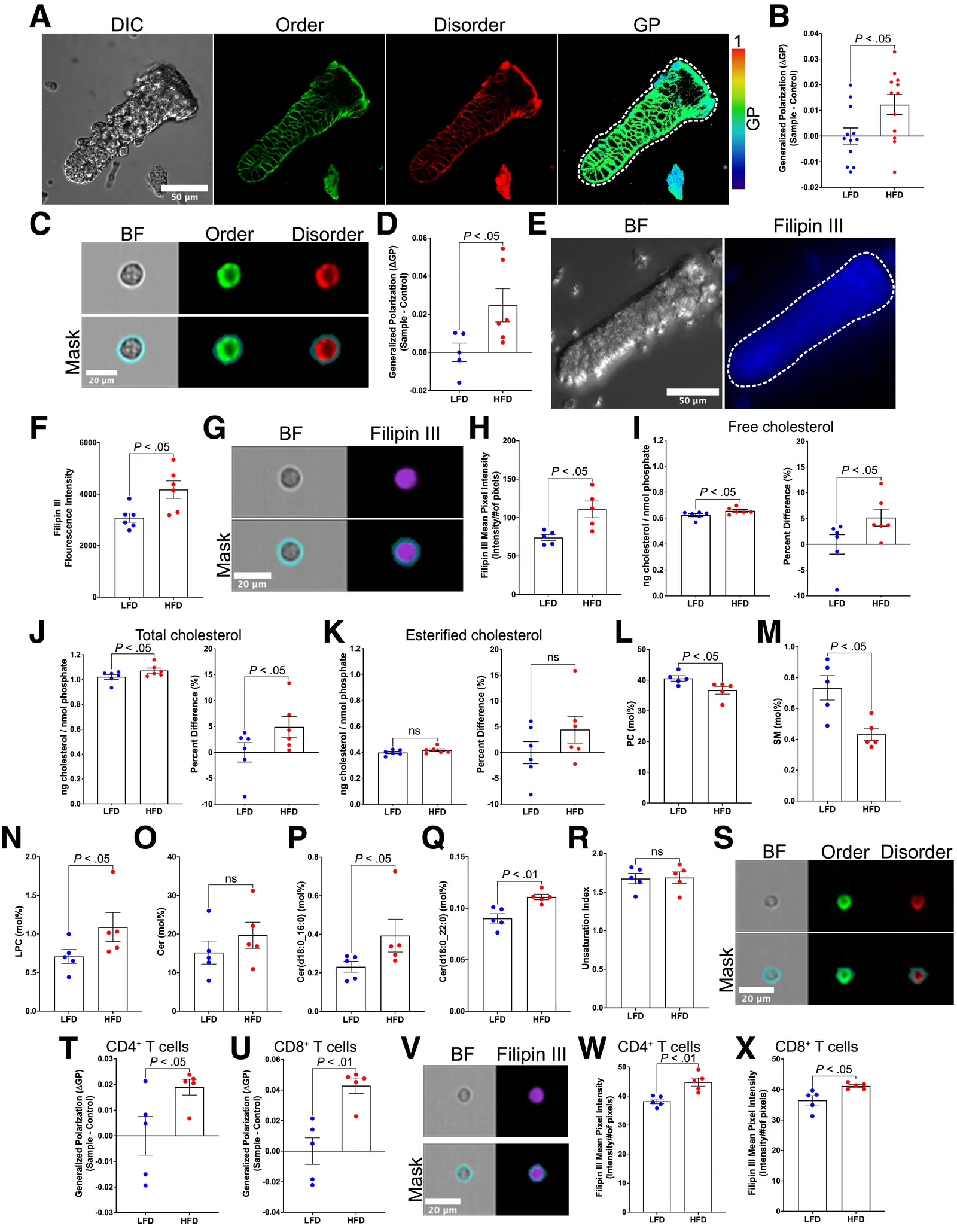
**HFD remodels colonic crypt lipid composition while increasing plasma membrane cholesterol and rigidity in colonocytes and splenic T cells.** (*A*) Representative confocal images with a region of interest (ROI) of mouse colonic crypts stained with Di4: DIC, order, disorder, and GP. (*B*) Quantification of membrane rigidity ΔGP (sample − control) values (n = 12 mice/diet). (*C*) Representative FlowSight images with plasma membrane mask of sorted Lgr5-GFP^+^ colonocytes stained with Di4: BF, order, and disorder. (*D*) Quantification of Lgr5-GFP^+^ plasma membrane rigidity ΔGP (sample − control) values (n = 5–6 mice/diet). (*E*) Representative widefield images with ROI of mouse colonic crypts stained with Filipin III. (*F*) Quantification of colonic crypt free cholesterol (n = 6 mice/diet). (*G*) Representative FlowSight images with plasma membrane mask of sorted Lgr5-GFP^+^ colonocytes stained with Filipin III. (*H*) Quantification of Lgr5-GFP^+^ stem/progenitor cell plasma membrane free cholesterol (n = 5 mice/diet). (*I*) Free cholesterol, (*J*) total cholesterol, and (*K*) cholesteryl ester abundance in lipid extracts from isolated colonic crypts measured using the Amplex Red Cholesterol Assay and normalized to lipid phosphate content. Lipidomic analysis of colonic crypts showing (*L*) PC mol%, (*M*) SM mol%, (*N*) LPC mol%, (*O*) Cer mol%, (*P*) Cer(d18:0_16:0) mol%, (*Q*) Cer(d18:0_22:0) mol%, and (*R*) phospholipid unsaturation index. (*S*) Representative FlowSight image of isolated splenic T cells stained with Di4. Quantification of splenic (*T*) CD4^+^ and (*U*) CD8^+^ T-cell plasma membrane rigidity ΔGP (sample − control) values (n = 5 mice/diet). (*V*) Representative FlowSight image of isolated splenic T cells stained with Filipin III. Quantification of splenic (*W*) CD4^+^ and (*X*) CD8^+^ T-cell plasma membrane free cholesterol (n = 5 mice/diet). Data are presented as mean ± standard error of the mean. Statistical significance between treatments (∗*P* ≤ .05, ∗∗*P* ≤ .01) was assessed by an unpaired (*I–K*) 1- or (*B, D, F, H, L–R, T–U* and *W–X*) 2-tailed Student *t* test. ns, not significant, *P* > .05. Scale bar (*A* and *E*), 50 μm and (*C, G, S* and *V*), 20 μm.

To further determine whether the increase in free cholesterol was accompanied by broader alterations in membrane lipid composition, we performed high-performance liquid chromatography-tandem mass spectrometry (HPLC-MS/MS)-based shotgun lipidomics on isolated colonic crypts. Among detected lipid classes, the abundance (mol%) of phosphatidylcholine (PC) and sphingomyelin (SM) was reduced in HFD samples, whereas lyso-phosphatidylcholine (LPC) was elevated (Figure 3*L–N*). Total ceramide (Cer) levels were not significantly different between groups; however, the abundance of specific species, including Cer(d18:0_16:0) and Cer(d18:0_22:0), were elevated in HFD-fed mice (Figure 3*O–Q*). The complete lipidomics dataset, including all detected lipid molecular species and fatty acid composition, is provided in Supplementary Table 1. Despite these class-specific changes, the overall membrane phospholipid unsaturation index^22^ was not different between the HFD and LFD groups (Figure 3R). Together, these data suggest that chronic HFD feeding does not broadly increase membrane fatty acid saturation but instead selectively remodels lipid composition, with cholesterol enrichment emerging as the dominant driver of increased membrane rigidity.

We subsequently assessed whether the diet-induced alterations to the plasma membrane were restricted to the intestinal epithelium. FlowSight-based analysis of splenic T cells revealed that HFD-fed mice also exhibited increased plasma membrane free cholesterol and rigidity in CD4^+^ and CD8^+^ T cells (Figure 3*S–X*), suggesting a systemic remodeling of membrane composition and structure. These findings align with previous reports linking dietary fat composition to alterations in plasma membrane rigidity and lipid raft–associated signaling in epithelial and immune cells.^23^, ^24^, ^25^, ^26^ Collectively, these findings identify plasma membrane free cholesterol accumulation as a previously underappreciated and dominant determinant of HFD-induced membrane rigidification across epithelial and immune compartments.

### A High-Fat Diet Remodels Membrane Receptor Nanoclusters and Activates β-Catenin Signaling

Because plasma membrane free cholesterol and rigidity have been linked to specialized signaling platforms (eg, protein condensates or nanoclusters),^27^, ^28^, ^29^, ^30^ we investigated how HFD modulates membrane-associated signaling in colonic stem cells. For this purpose, we utilized stochastic optical reconstruction microscopy (STORM) to assess the nanoscale organization of 2 critical receptors, EGFR and the Wnt co-receptor, LRP6, in colonic Lgr5-GFP^+^ cells from control and HFD-fed mice (Figure 4*A*). Super-resolution imaging revealed that HFD significantly increased the diameter of EGFR and LRP6 nanoclusters in colonic stem cells relative to controls (Figure 4*B* and *D*). Frequency distribution analysis showed a shift toward larger clusters, with a concurrent decrease in the relative abundance of smaller nanoclusters, indicating that HFD induced a reorganization of receptor assemblies at the plasma membrane (Figure 4*C* and *E*). Notably, larger receptor clusters have been shown to enhance downstream signaling efficiency by promoting the recruitment and retention of signaling effectors,^31^^,^^32^ suggesting a potential mechanism by which HFD may potentiate Wnt and EGFR signaling in ISCs. These observations are consistent with previous studies demonstrating that plasma membrane cholesterol content and rigidity directly modulate the nanoscopic clustering and activity of membrane receptors, including EGFR and Wnt coreceptors such as LRP6.^27^^,^^33^^,^^34^

**Figure 4:**
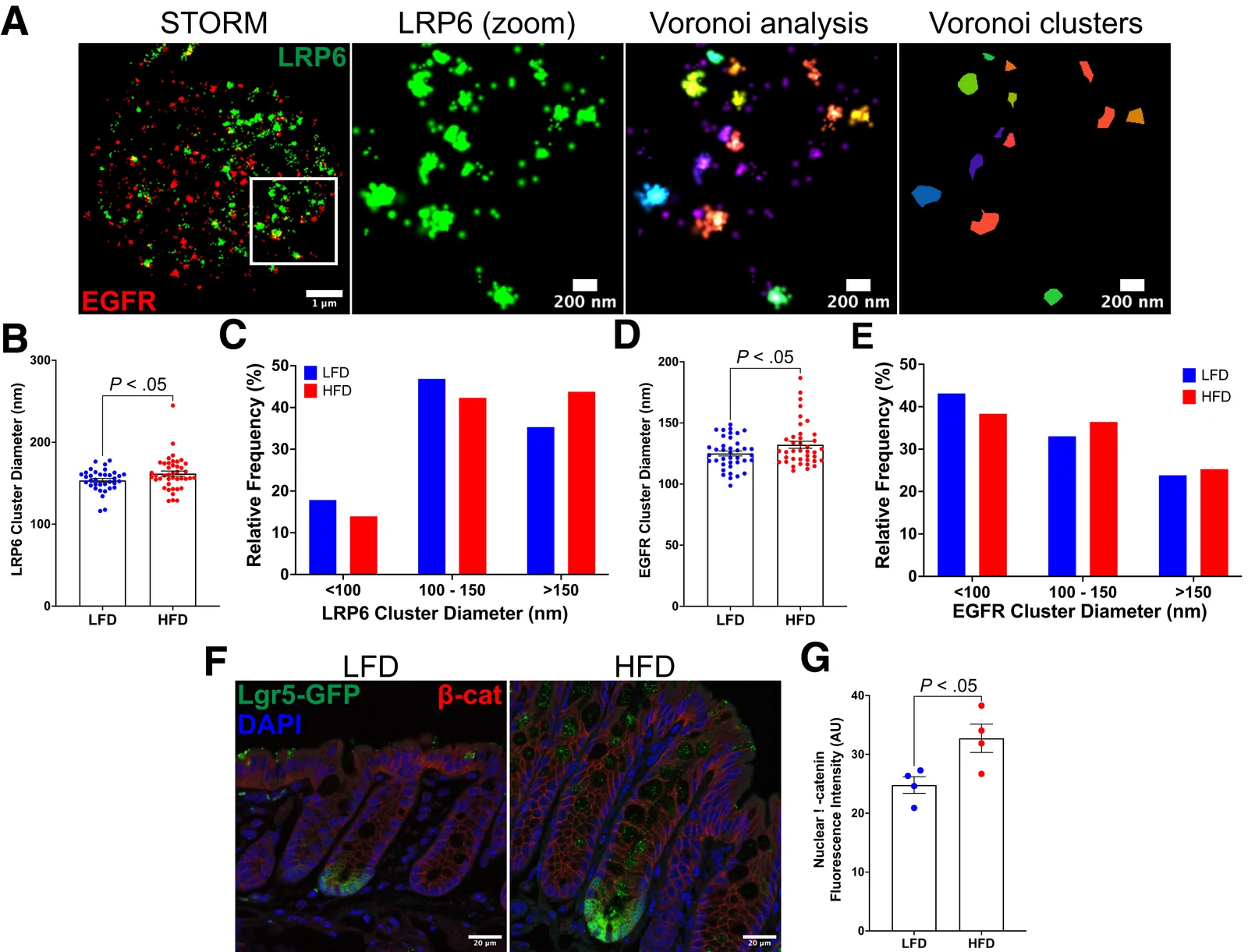
**HFD enhances membrane receptor nanoclusters and activates β-catenin signaling.** (*A*) Representative STORM images of mouse colonocytes stained with LRP6 antibodies and fluorescently labeled EGF ligand. Quantification of Lgr5-GFP^+^ (*B*) LRP6 nanocluster diameter (n = 35–42 fields of view [FOVs])/diet) and (*C*) LRP6 cluster diameter frequency (n = 5 mice/diet). Quantification of Lgr5-GFP^+^ (*D*) EGFR nanocluster diameter (n = 39 FOVs/diet) and (*E*) EGFR cluster diameter frequency (n = 5 mice/diet). (*F*) Representative immunofluorescence images of colon sections stained with 4′,6-diamidino-2-phenylindole (DAPI), Lgr5-GFP, and β-catenin from LFD- and HFD-fed mice. (*G*) Quantification of nuclear β-catenin accumulation (n = 4 mice/diet). Data are presented as mean ± standard error of the mean. Statistical significance between treatments was assessed by an unpaired 2-tailed Student *t* test, *P* > .05. Scale bar (*A*), 1 μm, (*A*, *zoom*) 200 nm, and (*F*), 20 μm.

To assess the functional consequences of plasma membrane receptor reorganization, we evaluated downstream β-catenin nuclear localization in Lgr5-GFP^+^ cells via immunohistochemistry (Figure 4*F*). Nuclear accumulation of β-catenin, a hallmark of canonical Wnt pathway activation, was markedly increased in crypt base stem and progenitor cells from HFD-fed mice compared with LFD control mice (Figure 4*G*), consistent with previous findings that HFD increases nuclear β-catenin in ISCs.^9^ These findings indicate that HFD-induced changes in plasma membrane composition and receptor organization enhance downstream Wnt–β-catenin signaling activity in colonic stem cells. To explore transcriptional changes associated with membrane homeostasis, we performed bulk RNA sequencing (RNA-seq) and Ingenuity Pathway Analysis (IPA) on colonic epithelial cells isolated from mice following 12 weeks of HFD feeding (Figure 5). Differential gene expression (DGE) analysis identified 13 significantly upregulated genes and 25 significantly downregulated genes in HFD-fed mice relative to LFD controls (Supplementary Table 2). Consistent with the relatively modest transcriptional response, canonical pathway analysis revealed limited pathway-level alterations, with only the Antigen Presentation Pathway (negative z-score) and the Zn Homeostasis Signaling Pathway (positive z-score) reaching IPA significance (Figure 5*B*). Interestingly, upstream regulator analysis revealed activation of cholesterol and membrane lipid regulatory factor, INSIG1, alongside signaling mediators linked to proliferation and epithelial adaptation (PTGS2-PGE2, SIRT1, and PIK3CG), coupled with suppression of immune and antigen-presentation pathways (CIITA, IFNG, and IL27) in HFD vs LFD control mice (Figure 5*C*). Notably, recent work has shown that HFD suppresses epithelial MHC II expression in intestinal stem cells and enhances tumor initiation, underscoring a potential link between diet-induced immune modulation and stemness.^35^ These coordinated transcriptional signatures are consistent with activation of lipid homeostatic and proliferative signaling programs and suppression of immune pathways.

**Figure 5:**
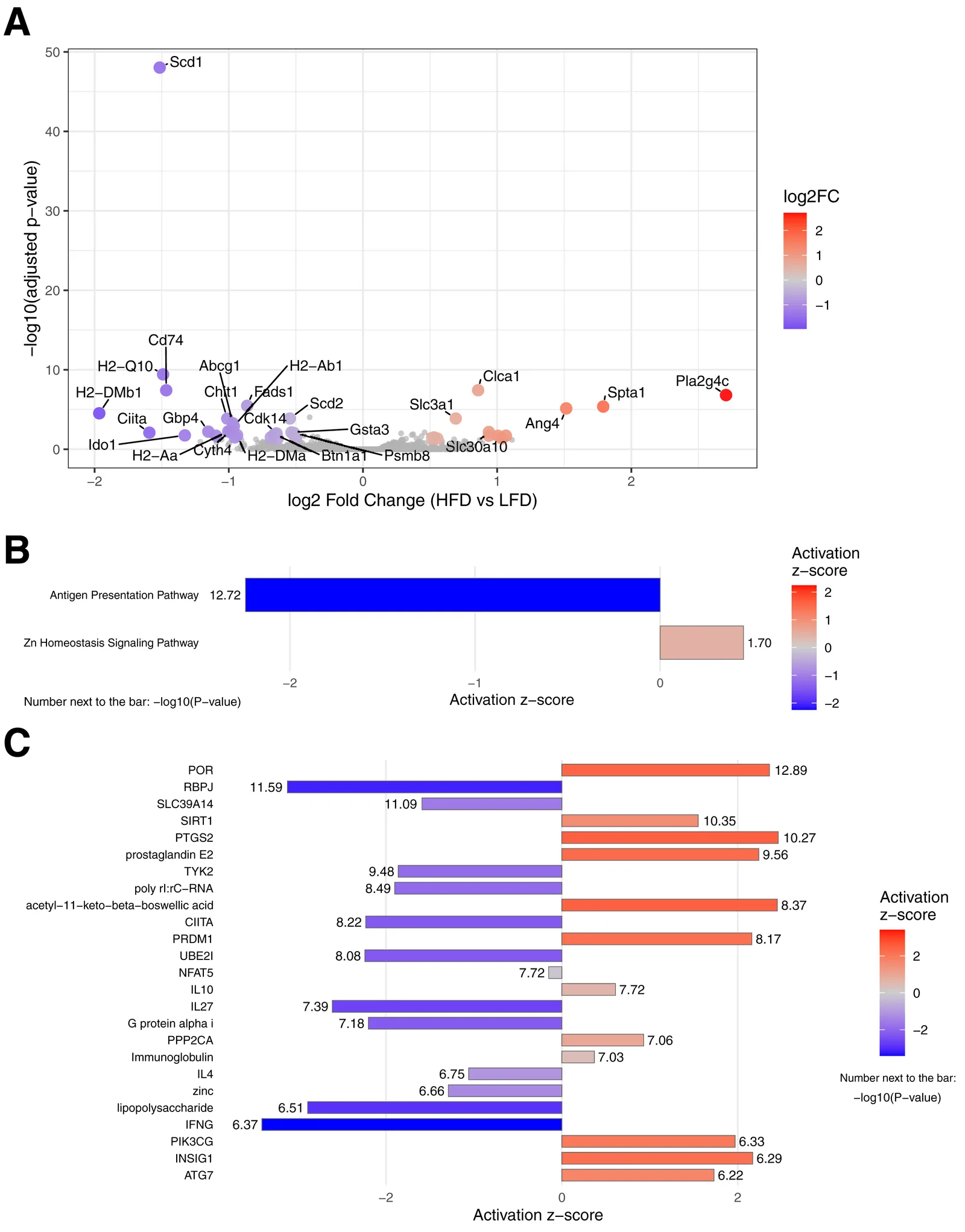
**IPA analysis of colonocyte bulk RNA-seq from 12-week HFD-fed mice.** (*A*) Volcano plot of DEGs (HFD vs LFD), n = 6 mice/diet (labeled top 25 genes). IPA (*B*) canonical pathway and (*C*) upstream regulator analyses highlighting lipid regulatory and immune-modulatory signals.

### Hyperphagia-Induced Obesity Does Not Recapitulate High-Fat Diet–Induced Membrane Remodeling

To determine whether membrane remodeling and receptor nanoclustering were driven by obesity or excess dietary fat exposure, we analyzed male leptin receptor–deficient (*db/db*) mice, which develop hyperphagic obesity on a standard LFD diet.^36^ Despite profound increases in body weight, serum cholesterol (total, high-density lipoprotein [HDL], and LDL) and fat mass by 11 weeks of age (Figure 6*A–I*), *db/db* mice, compared with *db/+* (control) mice, did not exhibit the membrane or signaling alterations observed in HFD-fed animals. Specifically, in contrast to HFD-fed mice, colonic crypts from *db/db* mice did not exhibit any significant difference in membrane rigidity compared with lean *db/+* controls, as measured by Di4 staining and confocal imaging (Figure 6*J*). Similarly, plasma membrane rigidity in colonocytes, imaged via FlowSight, was unchanged in *db/db* mice compared with *db/+* control mice (Figure 6*K*). Plasma membrane rigidity in splenic CD4^+^ and CD8^+^ T cells was also unaffected (Figure 6*L* and *M*). Filipin III staining revealed no significant increase in free cholesterol content in colonic crypts or in the plasma membranes of colonocytes or splenic T cells from *db/db* mice compared with *db/+* control mice (Figure 6*N–P*).

**Figure 6:**
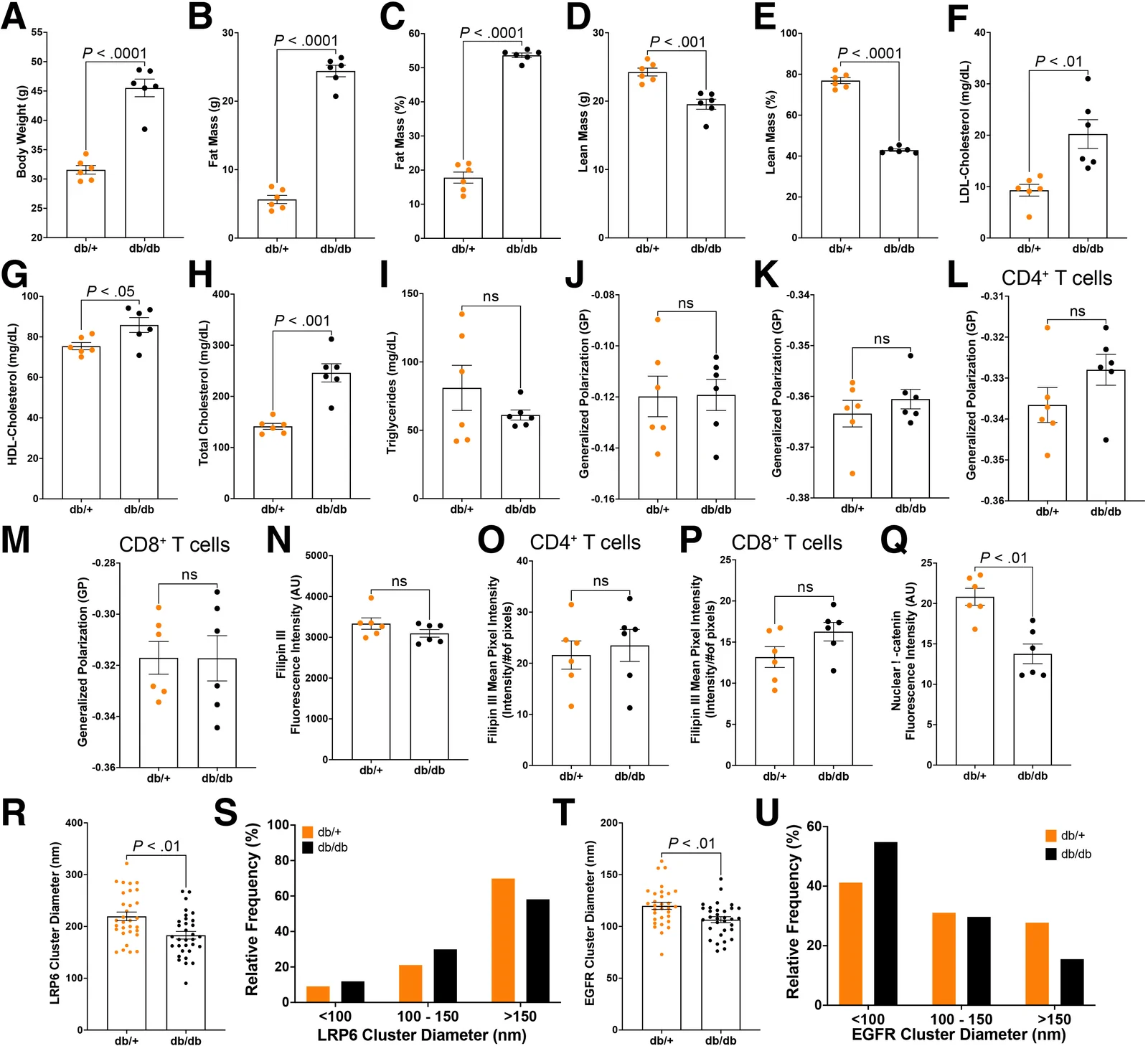
**Hyperphagia-induced obesity does not recapitulate HFD-induced membrane remodeling.** (*A*) Male mice body weight for *db/db* and *db/+* (control) mice. *Db/db* and *db/+* male mice (*B* and *C*) fat mass and (*D* and *E*) lean mass were measured via echoMRI (n = 6 mice/genotype). Quantification of serum (*F*) LDL cholesterol, (*G*) HDL cholesterol, (*H*) total cholesterol, and (*I*) triglycerides (n = 6 mice/genotype). Quantification of (*J*) colonic crypt membrane rigidity and (*K*) single-cell colonocyte plasma membrane rigidity GP values (n = 6 mice/genotype). Quantification of splenic (*L*) CD4^+^ and (*M*) CD8^+^ T cell plasma membrane rigidity ΔGP (sample − control) values (n = 6 mice/genotype). Quantification of (*N*) colonic crypt free cholesterol and splenic (*O*) CD4^+^ and (*P*) CD8^+^ T-cell plasma membrane free cholesterol (n = 6 mice/genotype). (*Q*) Quantification of nuclear β-catenin accumulation (n = 6 mice/diet). Quantification of colonocyte (*R*) LRP6 nanocluster diameter (n = 31–32 fields of view [FOVs]/diet) and (*S*) LRP6 cluster diameter frequency (n = 6 mice/diet). Quantification of colonocyte (*T*) EGFR nanocluster diameter (n = 31–32 FOVs/diet) and (*U*) EGFR cluster diameter frequency (n = 6 mice/diet). Data are presented as mean ± standard error of the mean. Statistical significance between treatments was assessed by an unpaired 2-tailed Student *t* test. ns, not significant, *P* > .05.

Super-resolution STORM microscopy revealed that the nanoscale organization of LRP6 receptors was altered in *db/db* colonocytes, but in the opposite direction of HFD-fed mice. Specifically, LRP6 nanocluster diameter was significantly reduced in *db/db* mice, with a relative enrichment of smaller clusters and a decrease in larger clusters (Figure 6*R* and *S*). EGFR nanoclustering was not significantly different in *db/db* mice compared with controls, though the distribution also trended toward smaller clusters (Figure 6*T* and *U*). These findings suggest that receptor nanoclustering is sensitive to membrane composition and organization, which appear to be influenced more by dietary fat than by obesity alone. Consistent with these membrane and receptor clustering phenotypes, nuclear accumulation of β-catenin was significantly reduced in colonic crypts from *db/db* mice compared with *db/+* controls (Figure 6*Q*). This reduction aligns with the absence of plasma membrane remodeling and receptor clustering that are thought to potentiate Wnt signaling.

To further contextualize the absence of membrane remodeling in *db/db* colonocytes, we performed bulk RNA-seq IPA analysis on colonic epithelial cells. DGE analysis identified 215 significantly upregulated genes and 127 significantly downregulated genes in *db/db* mice relative to *db/+* controls (Figure 7*A*; Supplementary Table 3). Canonical pathway analysis revealed enrichment of signaling programs associated with inflammation, stress response, and injury-related processes including endothelin-1 signaling, eicosanoid signaling, phospholipase activity, p38 MAPK, PAK, ILK, and S100 family signaling, pathways which are broadly linked to epithelial stress responses and innate immune activation rather than lipid-mediated membrane remodeling (Figure 7*B*). Consistent with this profile, upstream regulator analysis predicted activation of classical inflammatory and cytokine-driven mediators including IL1B, TNF, IL6, IFNG, TGFB1, HIF1A, and NF-κB, indicating a transcriptional environment dominated by immune and stress signaling rather than alterations in intracellular cholesterol trafficking, membrane lipid composition, or Wnt and β-catenin–potentiating pathways. Notably, regulators associated with lipid sensing and nuclear receptor-mediated signaling were predicted to be inhibited, including RXRA, RORA, and ADIPOQ, suggesting a relative suppression of lipid-response transcriptional programs (Figure 7*C*). Together, these transcriptomic signatures indicate that obesity driven by leptin receptor deficiency promotes inflammatory and stress pathways but does not activate the membrane-linked lipid regulatory or proliferative programs observed under HFD feeding conditions. Together, these results indicate that obesity induced by hyperphagia and leptin receptor deficiency does not reproduce the membrane biophysical remodeling, receptor nanoclustering, or β-catenin activation associated with the chronic consumption of HFD. A previous study indicates that intestinal adaptation to obesity in leptin receptor–deficient (*db/db*) mice is distinct from HFD-induced obese mice.^9^ This highlights the unique impact of dietary fat on epithelial membrane architecture and associated downstream signaling pathways, independent of body weight or adiposity.

**Figure 7:**
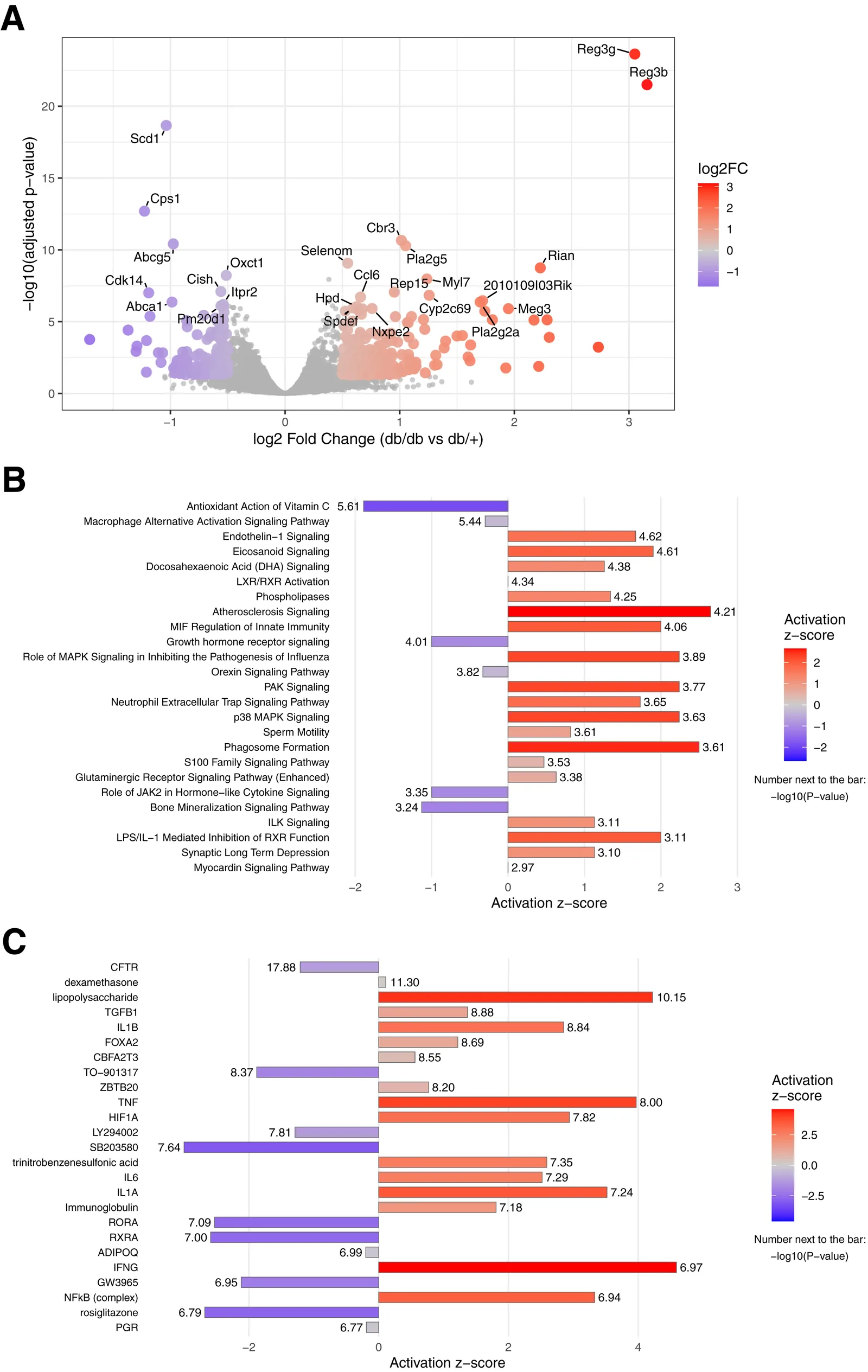
**IPA analysis of colonocyte bulk RNA-seq from *db/db* mice.** (*A*) Volcano plot of DEGs (*db/db* vs *db/+*), n = 6 mice/genotype (labeled top 25 genes). IPA (*B*) canonical pathway and (*C*) upstream regulator analyses highlighting inflammatory, stress-response, and immune-associated signaling programs.

### Acute High-Fat Diet (1 Week) Feeding is Sufficient to Remodel Colonocyte Plasma Membranes

To determine whether chronic HFD exposure is necessary to alter colonocyte membrane biophysics, we subjected mice to a short-term (1 week) HFD feeding regimen and assessed membrane properties in colonic crypts and colonocytes. Remarkably, plasma membrane rigidity, as measured by Di4 staining, was significantly increased in colonic crypts and isolated colonocytes from acutely fed HFD mice compared with LFD control mice (Figure 8*A* and *B*). Interestingly, this rapid remodeling occurred in the absence of any detectable changes in plasma membrane free cholesterol levels, as assessed by Filipin III staining in both colonic crypts and colonocytes (Figure 8*C* and *D*). These findings suggest that membrane rigidity is rapidly modulated during early HFD exposure.

**Figure 8:**
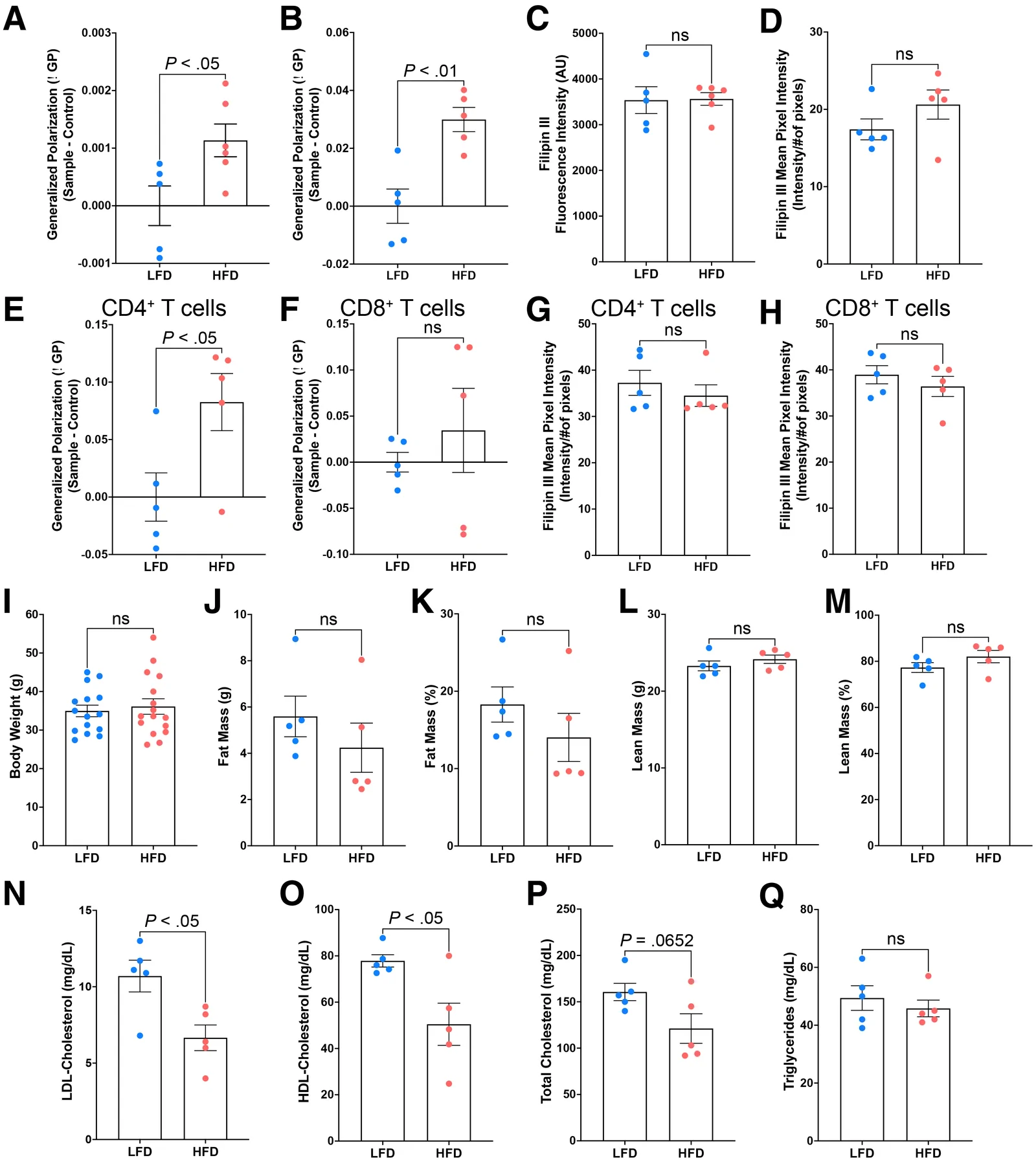
**Acute (1-week) HFD feeding is sufficient to remodel colonocyte and splenic CD4^+^ T cells plasma membrane rigidity.** Quantification of (*A*) colonic crypt membrane rigidity and (*B*) single-cell colonocyte plasma membrane rigidity ΔGP (sample − control) values (n = 5−6 mice/diet). Quantification of (*C*) colonic crypt free cholesterol and (*D*) single-cell colonocyte plasma membrane free cholesterol (n = 5−6 mice/diet). Quantification of splenic (*E*) CD4^+^ and (*F*) CD8^+^ T cell plasma membrane rigidity ΔGP (sample − control) values (n = 5 mice/diet). Quantification of splenic (*G*) CD4^+^ and (*H*) CD8^+^ T-cell plasma membrane free cholesterol (n = 5 mice/ diet). (*I*) Male mice body weight after 1 week on diet. Male mice (*J* and *K*) fat mass and (*L* and *M*) lean mass were measured via echoMRI at week 1 (n = 5 mice/diet). Quantification of serum (*N*) LDL cholesterol, (*O*) HDL cholesterol, (*P*) total cholesterol, and (*Q*) triglycerides (n = 5 mice/diet). Data are presented as mean ± standard error of the mean. Statistical significance between treatments was assessed by an unpaired 2-tailed Student *t* test. ns, not significant, *P* > .05.

To evaluate whether similar effects occurred in peripheral immune cells, membrane rigidity and cholesterol content in splenic CD4^+^ and CD8^+^ T cells was assessed. We observed a modest but significant increase in plasma membrane rigidity in splenic CD4^+^ T cells, whereas no significant changes were observed in splenic CD8^+^ T cells (Figure 8*E* and *F*). Free cholesterol levels were unchanged in both splenic T cell subsets (Figure 8*G* and *H*), indicating that acute HFD feeding selectively impacts membrane biophysics in immune cell populations. Previously, alterations in T cell membrane organization have been linked to changes in dietary lipid composition, especially the balance between saturated and polyunsaturated fatty acids.^37^

Despite clear alterations in membrane rigidity, no changes were observed in body weight, fat mass, or lean mass between control and HFD-fed mice after 1 week (Figure 8*I–M*), suggesting that membrane remodeling precedes overt metabolic phenotypes. Interestingly, mice acutely fed HFD exhibited reduced circulating levels of HDL- and LDL-cholesterol, whereas total cholesterol and triglyceride levels remained unchanged (Figure 8*N–Q*). These systemic lipid changes may reflect an early redistribution of lipid pools in response to HFD, potentially indicating initial homeostatic adaptations in intestinal lipid uptake and processing^38^^,^^39^ that appear diminished or altered after chronic HFD feeding.

To investigate the mechanisms driving early biophysical changes, we performed bulk RNA-seq and IPA analysis on colonocytes isolated after 1 week of HFD feeding. DGE analysis identified 306 significantly upregulated genes and 172 significantly downregulated genes following acute HFD-fed mice relative to LFD control (Figure 9*A*; Supplementary Table 4). Canonical pathway analysis revealed enrichment of signaling programs associated with early epithelial adaptation and growth factor responsiveness, including ERBB signaling, NGF-stimulated transcription, cell junction organization, and tumor microenvironment–associated pathways, along with enhanced glutamatergic receptor signaling and pathways involved in nutrient and ion transport (Figure 9*B*). These signatures are consistent with an early remodeling phase in which epithelial signaling networks respond to dietary fat, prior to overt metabolic or proliferative changes. Pathways related to cholesterol biosynthesis, cell cycle initiation, DNA replication, and O-linked glycosylation were predicted to be inhibited, suggesting that acute HFD feeding alters colonocyte signaling outputs prior to large-scale transcriptional reprogramming of lipid metabolism or proliferation (Figure 9*B*). Upstream regulator analysis further supported this interpretation, identifying activation of stress- and lipid-responsive mediators including INSIG1, POR, FOXO1, TP53, TNF, and PTEN, alongside predicted inhibition of regulators linked to proliferation and metabolism, including MYC and insulin signaling (Figure 9*C*). Together, these transcriptional signatures suggest that acute HFD exposure elicits a rapid epithelial homeostatic response characterized by early adjustments in cholesterol sensing, growth factor modulation, and stress adaptation, preceding the more extensive membrane remodeling, sterol accumulation, and proliferative reprograming observed under chronic HFD conditions.

**Figure 9:**
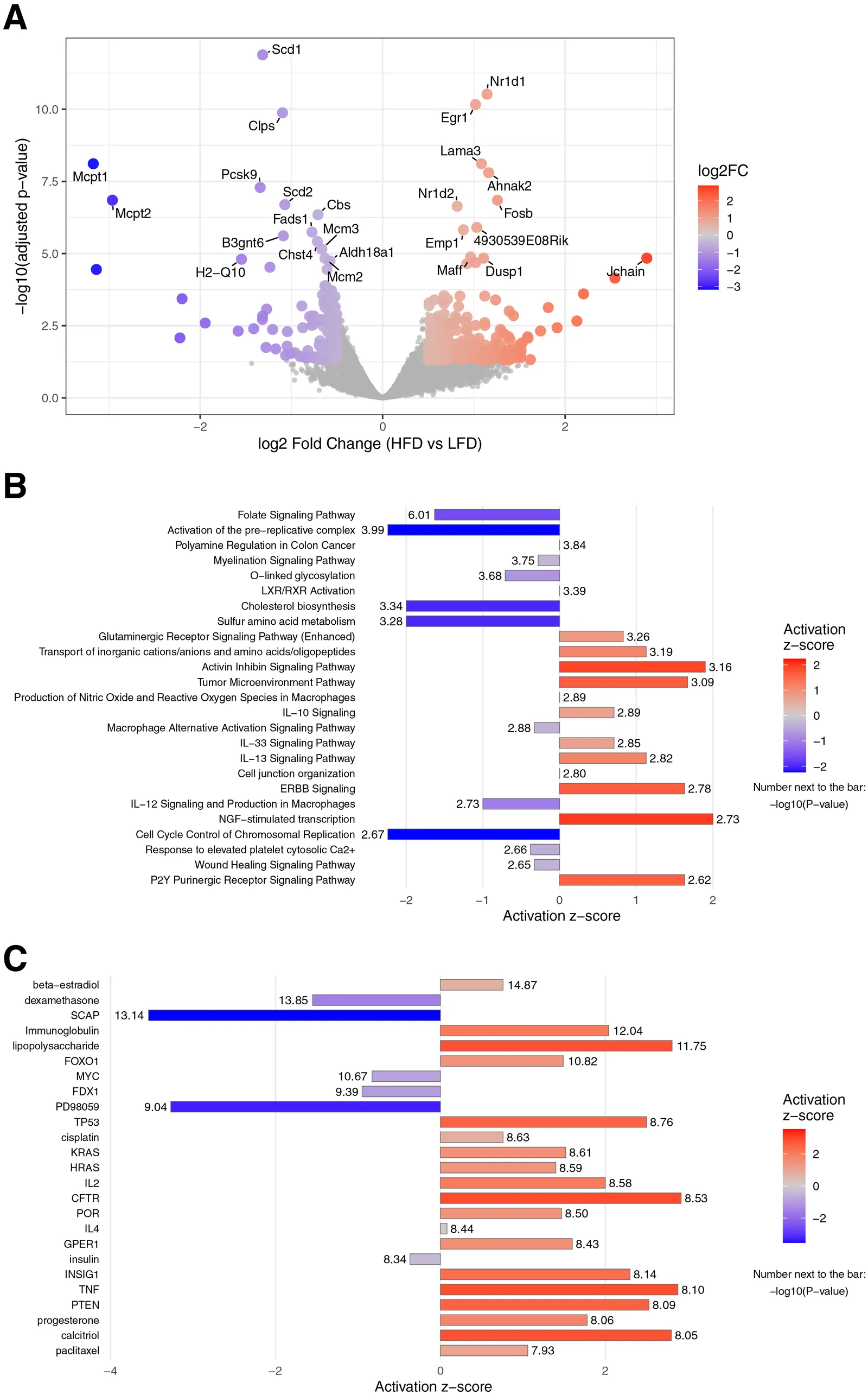
**IPA analysis of colonocyte bulk RNA-seq from 1-week HFD-fed mice.** (*A*) Volcano plot of DEGs (HFD vs LFD), n = 5 mice/diet (labeled top 25 genes). IPA (*B*) canonical pathway and (*C*) upstream regulator analyses highlighting early stress-adaptation, growth factor response, and immune-modulatory signaling.

### Low-Density Lipoprotein Cholesterol, but Not Palmitic Acid, Increases Membrane Rigidity In Vitro

Because HFD feeding mediates the accumulation of plasma membrane free cholesterol, thereby altering the membrane localization of key receptors that promote the overactivation of β-catenin and expansion of the ISC niche, we next determined whether exogenous cholesterol is sufficient to phenocopy the membrane biophysical changes observed in HFD-fed mice. For this purpose, we treated immortalized young adult mouse colonic epithelial cells (YAMCs) with exogenous human LDL cholesterol in lipoprotein-depleted fetal bovine serum for 24 hours. Image-based flow cytometry analyses revealed a dose-dependent increase in plasma membrane rigidity following LDL cholesterol treatment (Figure 10*A*), as assessed by Di4. Concurrently, there was a significant dose-dependent increase in plasma membrane free cholesterol, measured by Filipin III staining (Figure 10*B*). These results indicate that exogenous cholesterol is sufficient to biophysically remodel the colonocyte plasma membrane, similar to HFD exposure. Previously, cholesterol supplementation in mouse colonic organoids increased plasma membrane rigidity and free cholesterol levels, leading to a measurable increase in epithelial cell proliferation.^40^

**Figure 10:**
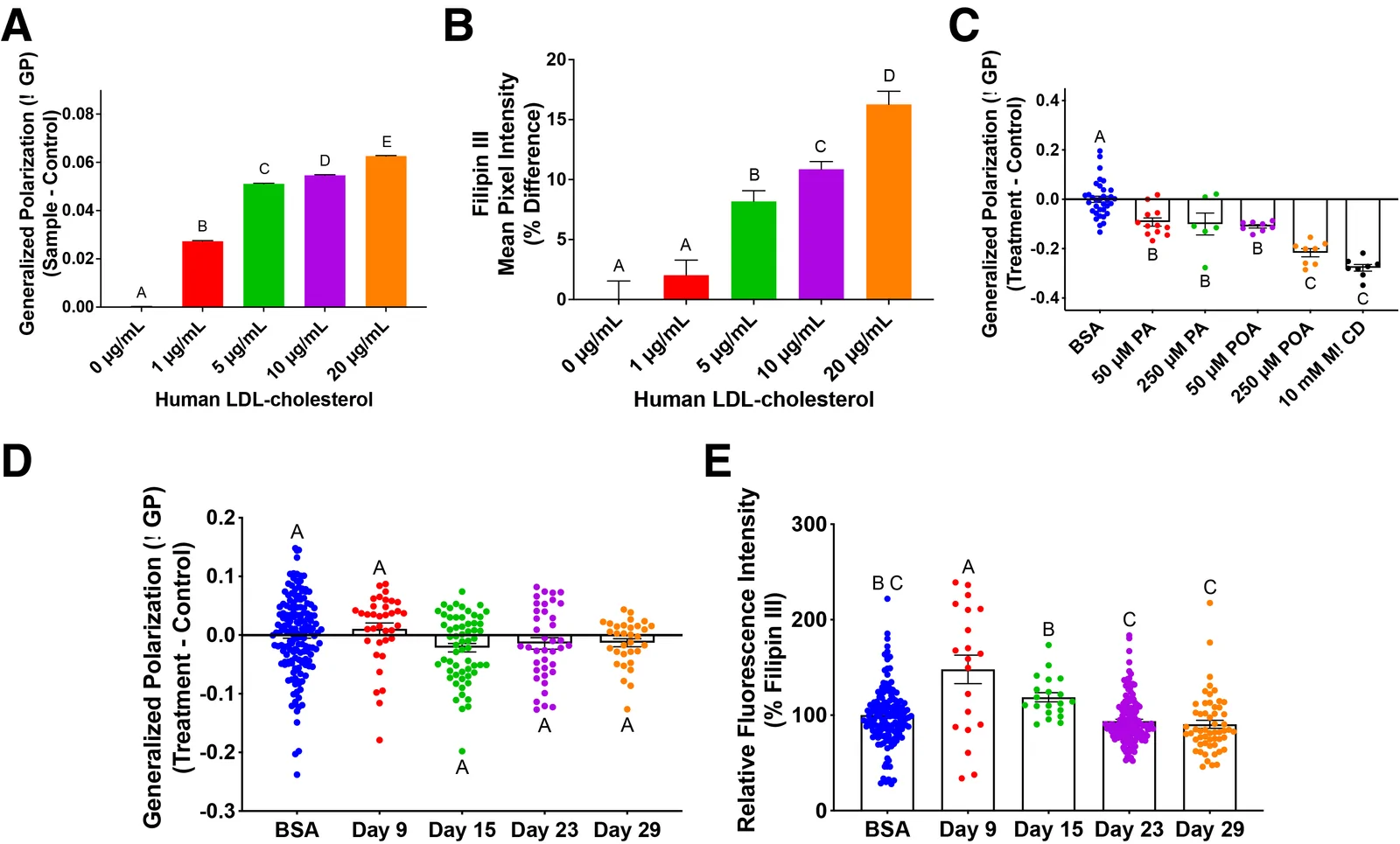
**LDL-cholesterol increases plasma membrane rigidity in vitro.** YAMC epithelial cells were grown in media containing lipoprotein-depleted fetal bovine serum (FBS) for 24 hours prior to being supplemented with varying doses of human LDL cholesterol for 24 hours. (*A*) Quantification of plasma membrane rigidity ΔGP (sample − control) values (n = 15,000–26,000 cells/treatment). (*B*) Quantification of plasma membrane free cholesterol (percent difference compared with control) (n = 2000–5000 cells/treatment). YAMC cells were treated with 50 or 250 μM PA or POA for 72 hours. Plasma membrane free cholesterol was depleted by incubating cells with 10 mM MβCD for 30 minutes at 37 °C (cholesterol-depleted control). Cells were then stained with Di4 and imaged by confocal microscopy. (*C*) Quantification of plasma membrane rigidity ΔGP (treatment − control) values (n = 6–34 fields of view [FOVs]/treatment). Human colonic organoids were treated with 30 μM of PA for up to 29 days. Culture media and PA were changed every other day. Intact organoids were isolated from Matrigel, stained with Di4 and imaged by confocal microscopy. (*D*) Quantification of membrane rigidity ΔGP (treatment − control) values (n = 32–160 organoids/treatment). Intact organoids were fixed in 4% PFA then stained with Filipin III and imaged by wide-field microscopy. (*E*) Quantification of normalized membrane free cholesterol (n = 20–155 organoids/treatment). Data are presented as mean ± standard error of the mean. Statistical significance between treatments as indicated by different letters (*P* ≤ .05) was assessed by Tukey’s multiple comparisons *t* test.

To explore whether fatty acids might similarly modulate membrane properties, we exposed YAMCs to 50 or 250 μM of either palmitic acid (PA; C16:0), a saturated fatty acid enriched in HFD, or palmitoleic acid (POA; C16:1), a monounsaturated fatty acid, for 72 hours. In contrast to the effects of cholesterol, both PA and POA treatments significantly decreased membrane rigidity compared with bovine serum albumin (BSA) controls (Figure 10*C*). Treatment with 10 mM methyl-β-cyclodextrin (MβCD), a cholesterol-depleting agent, served as a positive control for membrane fluidization and likewise decreased membrane rigidity. To examine the effects of long-term fatty acid exposure in a more physiologically relevant system, we also treated human colonic organoids with 30 μM PA for up to 29 days and examined membrane properties at days 9, 15, 23, and 29. No changes in plasma membrane rigidity were observed at any timepoint (Figure 10*D*). However, Filipin III staining revealed a transient increase in membrane free cholesterol at days 9 and 15 that normalized by days 23 and 29 (Figure 10*E*). These results suggest that, although PA may transiently increase membrane cholesterol, it is not sufficient to induce sustained membrane rigidification. These findings are consistent with a previous report demonstrating that PA as well as oleic acid (C18:1) treatment produced only minimal lipidomic changes.^22^ Overall, these data demonstrate that cholesterol loading, but not fatty acid exposure alone, is sufficient to induce membrane rigidification in colonic epithelial cells. This supports the hypothesis that the membrane remodeling phenotype observed in HFD-fed mice may be primarily mediated by dietary cholesterol rather than by saturated and monounsaturated fatty acids alone.

### Single-Cell Analysis Reveals High-Fat Diet–Induced Stemness and Cell-Cycle Activation in Intestinal Stem Cells

To address how chronic HFD promotes Lgr5^+^ stem cell expansion and membrane remodeling, we performed single-cell RNA sequencing (scRNA-seq) on cells dissociated from isolated colonic crypts following 12 weeks of HFD or LFD feeding. Colonocytes from 3 mice per condition were pooled prior to 10X Genomics library generation. The data captured transcriptional cell–state differences between dietary conditions but did not permit assessment of interanimal variability or diet-associated changes in cell-type abundance. Cell-resolved transcriptional features were examined across epithelial populations and within the ISC compartment. Unsupervised clustering identified 7 colonocyte subtypes (eg, ISCs, transit-amplifying cells, absorptive progenitor cells, absorptive colonocytes, goblet cells, enteroendocrine cells, and tuft cells), as well as a small immune cell population (Figure 11*A*). Cluster identities were supported by established lineage markers summarized in a dot plot (Figure 11*B*); however, we did not observe differences in the relative proportion of colonocyte cell types between HFD and LFD control mice (Figure 11*C*). Although differences in cell cycle phase estimation (Figure 11*D*) were not observed across the entire colonocyte compartment, HFD feeding increased the expression of canonical stem-and proliferation-associated transcripts including *Lgr5* and *Mki67* (Figure 11*E*), consistent with flow cytometry, EdU labeling, and organoid data (Figure 1). In parallel, a cell Correlation of Connectome and Transcriptome potency analysis utilizing a single-cell potency framework^41^ indicated higher potency scores in HFD verses LFD colonocytes (Table 2), suggesting a global shift toward less differentiated or more stem-like transcriptional states. Given the centrality of stem cells to colorectal tumor initiation, we next focused on ISCs. Within the ISC cluster, HFD was associated with increased expression of *Lgr5* and the proliferation marker *Mki67*, together with coordinated upregulation of mitotic and stem and progenitor genes including *Foxm1*, *Birc5*, *Stmn1*, *Top2a*, *Ube2c*, and the Wnt effector *Tcf4* (Figure 11*F*). Conversely, HFD ISCs exhibited reduced expression of short chain fatty acid transporters linked to epithelial fuel handling, including *Slc16a1* (MCT1) and *Slc5a8* (SMCT1), as well as *Lpcat2*, an acyltransferase that can influence phospholipid remodeling and membrane composition (Figure 11*G*). Consistent with a shift toward a more stem-like transcriptional state, cell potency Correlation of Connectome and Transcriptome scores were also elevated within HFD ISCs relative to LFD ISCs (Table 2). To further probe these transcriptional shifts at the pathway level, we applied UCell signature scoring^42^ to MSigDB gene sets. HFD ISCs displayed higher scores for Wnt pathway modules including Signaling by Wnt (REACTOME), Tcf-Dependent Signaling in Response to Wnt (REACTOME), and β-catenin–independent Wnt Signaling (REACTOME), alongside increased Cell Cycle (REACTOME), Myc Targets V1 (HALLMARK), Myc Targets V2 (HALLMARK), and E2F Targets (HALLMARK) programs (Supplementary Table 5). UCell signatures related to membrane-associated organization were also elevated in HFD ISCs, including Protein Localization to Membrane Rafts (Gene Ontology Biological Process [GOBP]) and Protein Transport into Membrane Rafts (GOBP) (Supplementary Table 5), consistent with our imaging findings (ie, increased plasma membrane receptor nanoclustering) (Figure 4). Metabolic and stress-adaptation programs were concurrently increased in HFD ISCs, including Glycolysis (HALLMARK), Oxidative Phosphorylation (HALLMARK), Aerobic Respiration (GOBP), mTORC1 Signaling (HALLMARK), PI3K-AKT-mTOR Signaling (HALLMARK), Reactive Oxygen Species Pathway (HALLMARK), and Unfolded Protein Response (HALLMARK) pathways (Supplementary Table 5), all features consistent with a proliferative, biosynthetically demanding ISC state. Additionally, HFD ISCs exhibited lower UCell scores for Hedgehog Signaling (HALLMARK), TNFα Signaling via NF-κB (HALLMARK), Transforming Growth Factor β Receptor Signaling Pathway (GOBP), Intracellular Glucose Homeostasis (GOBP), and Short Chain Fatty Acid Catabolic Process (GOBP) (Supplementary Table 5), suggesting diet-associated remodeling of niche and metabolic signaling axes relevant to stemness regulation.

**Figure 11:**
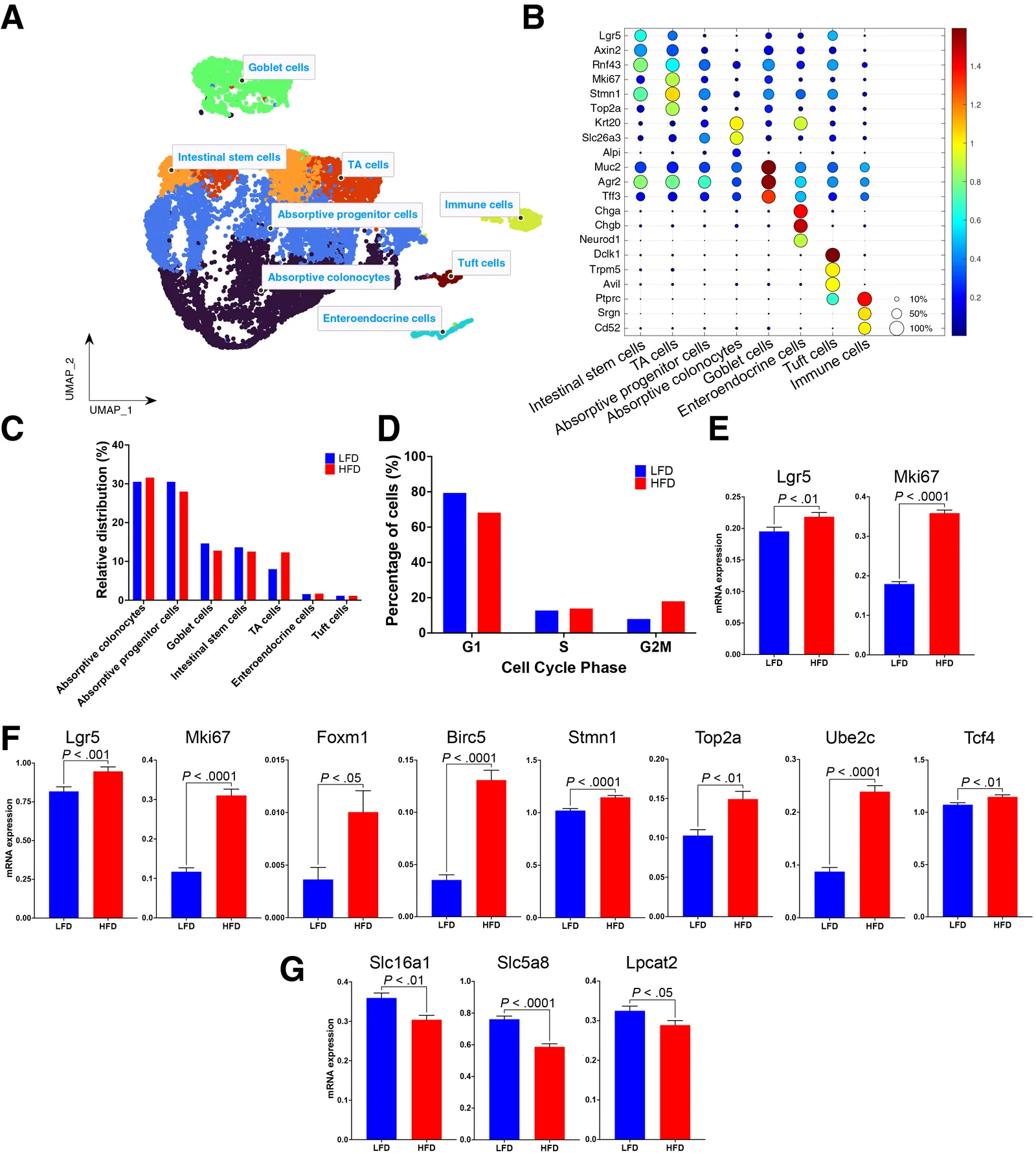
**HFD promotes Wnt–β-catenin and proliferation in intestinal stem cells.** (*A*) Uniform Manifold Approximation and Projection (UMAP) of colonocyte subtype populations and immune cells. (*B*) Dot plot depicting colonocyte subtype-specific marker genes (*dot color* represents normalized gene expression level and *dot size* represents the percentage of cells expressing the indicated gene). (*C*) Relative cell type distribution. (*D*) Cell–cycle phase distribution. (*E*) Normalized colonocyte gene expression of *Lgr5* and *Mki67*. (*F* and *G*) Normalized intestinal stem cell gene expression. Data are presented as mean ± standard error of the mean. Statistical significance between treatments was assessed by Mann–Whitney test, *P* > .05.

**Table 2:** Cell Differentiation Potency (CCAT) Scores

Cell types	LFD CCAT scores		HFD CCAT scores		*P* value
	Mean ± SD	No. cells	Mean ± SD	No. cells	
All colonocytes	0.1887 ± 0.0412	5965	0.1890 ± 0.0454	6708	<.05
ISCs	0.2160 ± 0.0146	813	0.2186 ± 0.0161	840	<.001

NOTE. Cell differentiation potency (CCAT) scores for all colonocytes and ISCs.

CCAT, Correlation of Connectome and Transcriptome; HFD, high-fat diet; ISC, intestinal stem cell; LFD, low-fat diet; SD, standard deviation.

Because our central premise links HFD to the promotion of cholesterol-driven plasma membrane remodeling, we next examined sterol and cholesterol-related signatures in our single-cell transcriptomic data set. Notably, HFD ISCs exhibited an increase in UCell enrichment scores for sterol and cholesterol biosynthesis including Sterol Biosynthetic Process (GOBP) and Cholesterol Biosynthesis (REACTOME), together with reduced scores for multiple homeostatic handling programs, including Cholesterol Efflux (GOBP), Cholesterol Storage (GOBP), Reverse Cholesterol Transport (GOBP), Sterol Homeostasis (GOBP), and Cholesterol Homeostasis (HALLMARK) (Supplementary Table 5). Genes linked to this pattern included increased expression of core biosynthetic- and mevalonate-associated transcripts (*Cyp51*, *Ebp*, *Fdps*, *Idi1*, *Mvk*, *Pmvk*) and *Pcsk9*. Although these transcript-level changes do not directly measure cholesterol flux, the combination of increased biosynthetic programs with reduced homeostatic-response signatures is consistent with perturbed sterol handling that could favor free cholesterol accumulation, consistent with our Filipin III and Di4 measurements in Lgr5^+^ cells (Figure 3). DGE analysis of ISCs identified 203 significantly upregulated genes and 87 significantly downregulated genes in HFD-fed mice relative to LFD control (Supplementary Table 6), further supporting a dominant proliferative shift following chronic consumption of HFD. Among the most strongly induced transcripts were mitotic regulators (*Ube2c*, *Ccnb1*, *Ccnb2*, *Cdc20*, *Plk1*, *Birc5*), whereas comparatively fewer genes were significantly downregulated (Figure 12*A*). Consistent with this finding, pathway enrichment (Enrichr) of upregulated differentially expressed genes (DEGs) identified REACTOME terms strongly centered on cell–cycle progression, checkpoint control, spindle and kinetochore signaling, and sister chromatid cohesion (Figure 12*B*). In addition, consistent with the expanded and hyperproliferative stem cell phenotype observed under chronic HFD feeding, IPA analysis of single-cell transcriptomes from ISCs revealed strong enrichment of mitotic and cell–cycle-associated programs, including mitotic prometaphase, kinetochore metaphase signaling, cell-cycle checkpoints, mitotic G2-G2/M phases, polo-like kinase signaling, and kinesin-mediated mitotic processes (Figure 12*C*). These signatures align with increased expression of proliferation-associated genes and elevated cell potency scores observed in HFD ISCs. Upstream regulator analysis further supports this proliferative state, predicting activation of key transcriptional and signaling regulators linked to intestinal stem cell self-renewal and growth factor responsiveness, including FOXM1, TCF4, and ERBB2, as well as predicted inhibition of cell-cycle restraint and stress-associated regulators CDKN1A and TP53 (Figure 12*D*). Notably, activation of LDLR and growth factor signaling nodes mirrored the lipid-responsive and proliferative upstream regulator signatures observed in bulk RNA-seq analysis from 12-week HFD colonocytes, indicating shared transcriptional features between the 2 datasets. Together, these data are consistent with the membrane remodeling and enhanced Wnt and EGFR signaling phenotypes detected by imaging and functional assays (Figure 2, Figure 3, Figure 4) and affirm that chronic HFD feeding drives a robust mitotic and stemness-associated transcriptional program within ISCs. In addition, our findings reinforce the functional and biophysical evidence that membrane remodeling under HFD conditions supports sustained ISC proliferation and activation.

**Figure 12:**
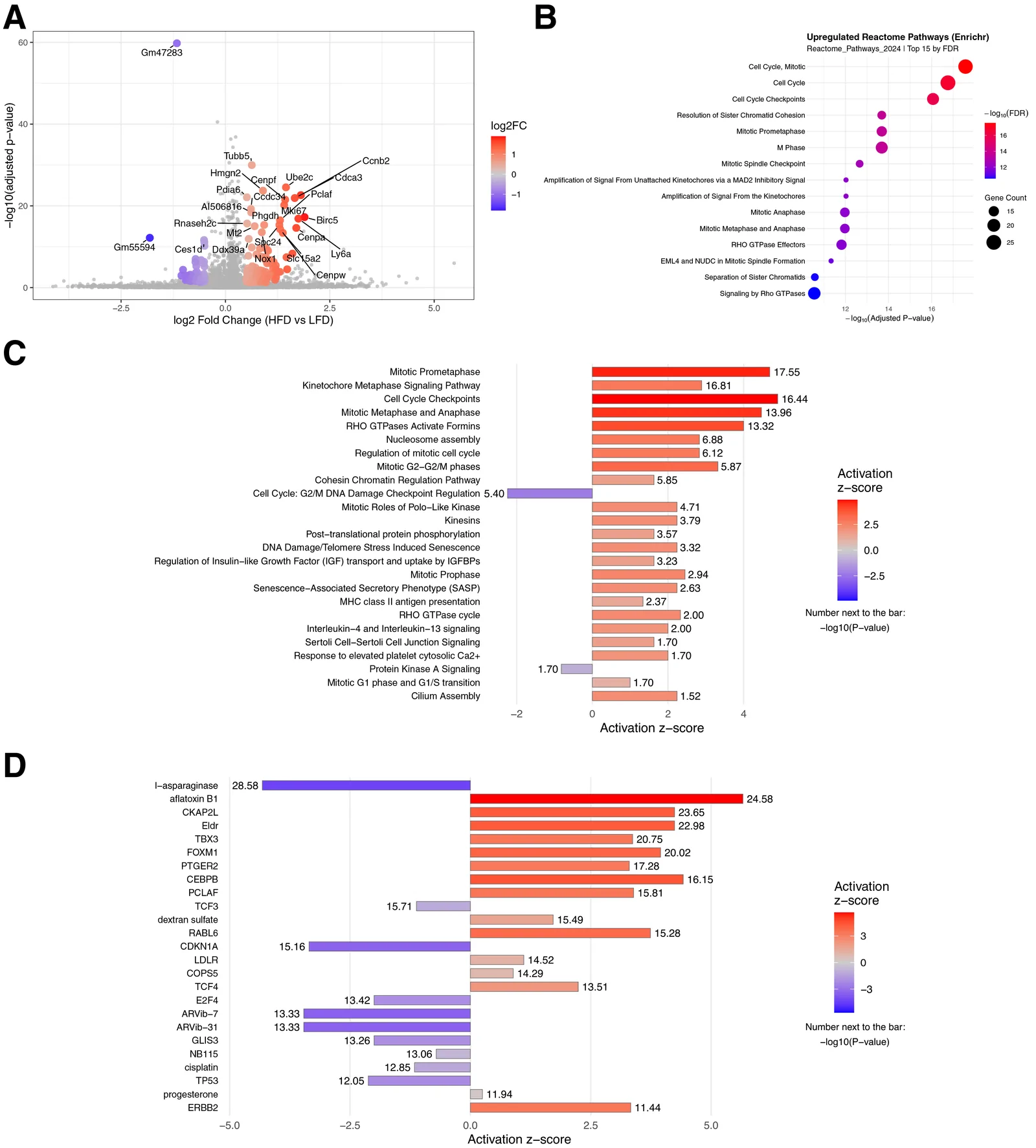
**IPA analysis of ISC scRNA-seq from 12-week HFD-fed mice.** (*A*) Volcano plot of differentially expressed genes (HFD vs LFD) n = 3 mice pooled/treatment (labeled top 25 genes). (*B*) Gene enrichment (Enrichr) analysis of the upregulated DEGs (showing top 15 pathways). IPA (*C*) canonical pathway and (*D*) upstream regulator analyses highlighting proliferative, mitotic, and growth factor transcriptional programs.

## Discussion

Diet and lifestyle are well-established modifiable risk factors for CRC development,^1^ yet the upstream driving mechanisms by which dietary lipid exposure reshapes intestinal stem cell behavior remain incompletely understood. This is noteworthy, because rapidly cycling Lgr5^+^ stem cells are exquisitely sensitive to dietary factors that modulate colon cancer risk^8^ and are considered the cells of origin of intestinal cancer.^6^ In this study, we identify plasma membrane remodeling as a previously underappreciated, diet-sensitive mechanism linking HFD exposure to enhanced colonic Lgr5^+^ stem cell function. Specifically, chronic HFD feeding modulates membrane biochemical and biophysical properties by increasing plasma membrane cholesterol content and rigidity in colonic epithelial cells, promoting nanoscale reorganization of key signaling membrane receptors, and enhances Wnt- and β-catenin-dependent colonic stem cell proliferation (Figure 13). Consistent with this interpretation, unbiased HPLC-MS/MS lipidomic profiling revealed selective remodeling of colonocyte lipid classes without a global shift in membrane phospholipid saturation. Although PC and SM abundance were reduced and LPC increased, the overall membrane phospholipid unsaturation index remained unchanged. These findings indicate that the increased rigidity observed under chronic HFD conditions is not driven by bulk fatty acyl saturation changes but rather by cholesterol enrichment and selective lipid redistribution. Importantly, biochemical cholesterol measurements revealed that free and total cholesterol abundance levels were increased, whereas there were no detectable differences in cholesteryl ester levels, suggesting that HFD expands the membrane-accessible free cholesterol and total cholesterol pools while maintaining relatively similar esterified cholesterol levels. This reinforces cholesterol as a principal biophysical determinant of membrane rigidity and aligns with prior work showing that phospholipid remodeling and cholesterol availability directly modulate intestinal stem cell homeostasis and tumorigenesis.^13^ Importantly, these effects are driven by HFD exposure rather than obesity per se and can be recapitulated by exogenous cholesterol loading in vitro. Notably, although previous studies demonstrated that HFD exposure and dietary cholesterol enhance intestinal stem cell function and tumorigenic potential,^9^^,^^13^ the present study identifies a distinct upstream biophysical mechanism. Specifically, we show for the first time that HFD rapidly remodels plasma membrane free cholesterol content, rigidity, and receptor nanoclustering, thereby perturbing colonic stem cell signaling.

**Figure 13:**
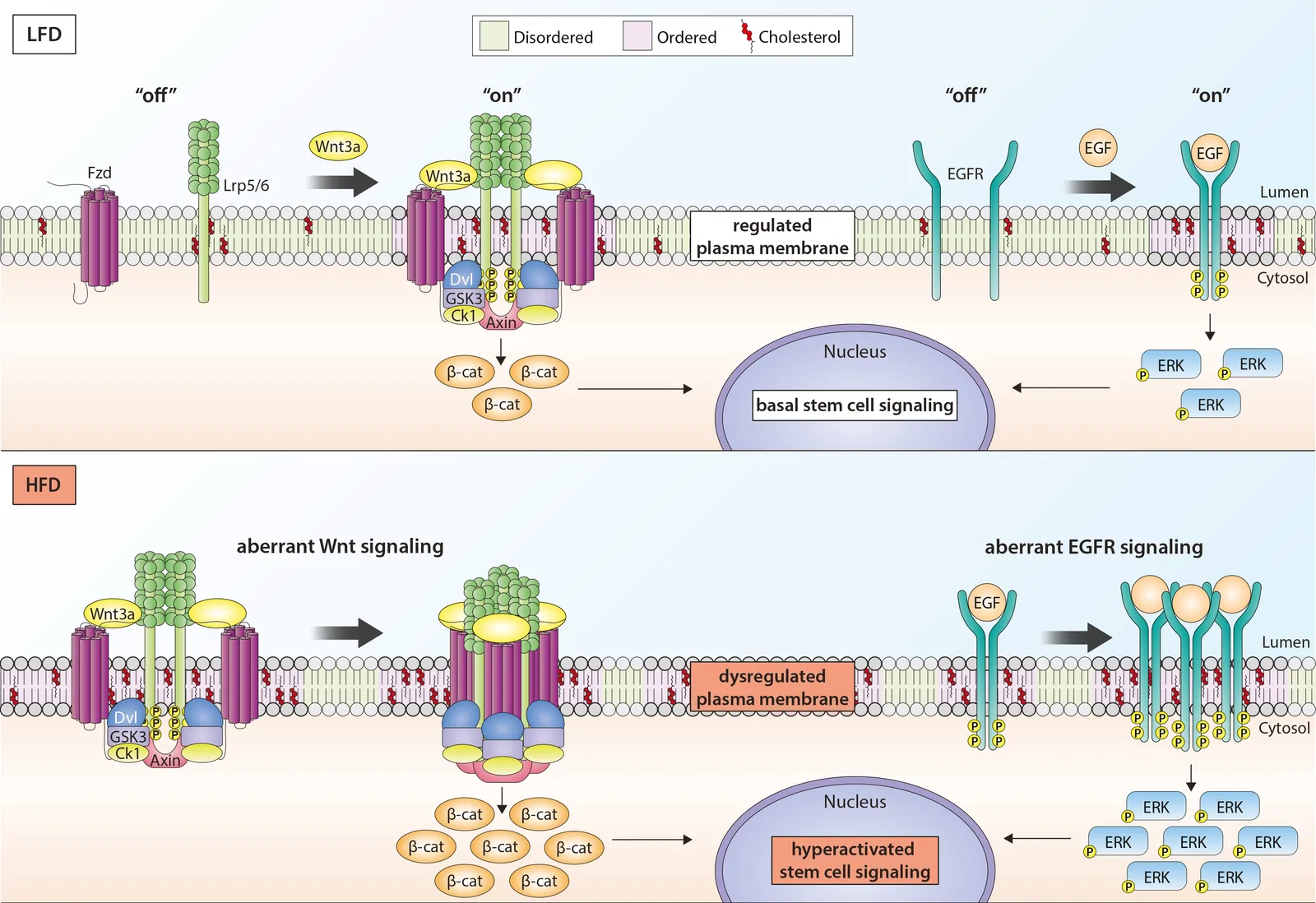
**Link between chronic HFD feeding and the loss of plasma membrane homeostasis in colonic stem cells.** HFD drives aberrant signaling by increasing plasma membrane free cholesterol and rigidity, thereby promoting Wnt and EGFR nanoclustering and subsequent downstream signaling and cell proliferation.

Chronic HFD feeding increased crypt length, Lgr5^+^ stem cell frequency and proliferation, and organoid-forming capacity, consistent with prior reports linking dietary fat to ISC expansion and tumor susceptibility.^9^^,^^11^^,^^43^ These include studies demonstrating that HFD and high cholesterol diets exacerbate azoxymethane-induced tumor initiation and progression, supporting the concept that dietary lipids sensitize the epithelium to oncogenic insults.^11^^,^^44^ Importantly, our study extends these observations by identifying membrane cholesterol accumulation and increased membrane rigidity as upstream biophysical and structural alterations that perturb downstream cellular responses. Using polarity-sensitive dyes and image-based flow cytometry, we demonstrate that these membrane alterations occur specifically at the plasma membrane and are evident in Lgr5^+^ stem cells, implicating the plasma membrane as an active regulatory interface rather than a passive structural component.

Super-resolution imaging revealed that HFD-induced membrane remodeling is accompanied by the reorganization of EGFR and LRP6 into larger nanoclusters. This is noteworthy, because nanoscale receptor clustering has been shown to enhance signaling efficiency by stabilizing ligand–receptor interactions and prolonging downstream signal propagation.^45^^,^^46^ Prior work has established nanoclustering as a dominant organizing principle of the plasma membrane, with cholesterol-enriched domains acting as platforms for signal amplification.^47^ In the context of ISCs, Wnt coreceptors, as well as EGFR, preferentially signal within rigid, cholesterol-rich membrane domains.^17^^,^^27^ Our observation that HFD increases membrane receptor cluster size while reducing the relative abundance of smaller nanoclusters suggests a shift toward signaling configurations that favor sustained pathway activation. Consistent with this model, HFD-fed mice exhibited increased nuclear β-catenin accumulation within crypt base stem and progenitor cells, consistent with enhanced canonical Wnt signaling. This framework is supported by prior studies showing that cholesterol selectively facilitates canonical Wnt pathway activation and promotes assembly of signaling-competent receptor complexes.^48^ Notably, the membrane and signaling phenotypes share conceptual parallels with mutant APC-driven epithelial transformation, where dysregulated Wnt signaling, altered membrane organization, and enhanced receptor signaling cooperatively drive ISC expansion and tumor initiation.^33^ Together, these findings support a model in which chronic HFD feeding increases free cholesterol levels in the plasma membrane, driving the reorganization of EGFR and LRP6 nanoclusters and enhancing β-catenin nuclear translocation and transcriptional activation in colonic stem cells. These coordinated membrane-centric and downstream transcriptional changes provide a mechanistic basis for the observed expansion of the intestinal stem cell pool and increased proliferative capacity in the HFD colon.

Surprisingly, genetic obesity, driven by leptin receptor deficiency, failed to recapitulate the membrane, receptor, or signaling phenotypes, despite marked increases in body weight and circulating cholesterol. In *db/db* vs control (*db*/+) mice, membrane rigidity and cholesterol content in colonocytes remained unchanged, receptor nanoclusters shifted toward smaller sizes, and nuclear β-catenin levels were reduced. Consistent with our findings, a prior study comparing dietary and genetic models of obesity reported that intestinal adaptation in *db/db* mice was largely opposite to that observed in chronic HFD-fed animals,^9^ and HFD-dependent suppression of epithelial MHC-II has likewise been reported to be diet-driven rather than reproduced in *db/db* mice,^35^ underscoring the distinct epithelial responses to dietary lipid exposure vs obesity per se. Importantly, obesity-associated CRC risk is multifactorial and may arise through mechanisms including chronic inflammation, hyperinsulinemia, altered adipokine signaling, microbiome dysbiosis, and metabolic dysfunction. Therefore, increased CRC risk in obesity does not necessarily require enhanced epithelial Wnt signaling at baseline. Furthermore, leptin receptor deficiency produces metabolic and signaling alterations that differ substantially from diet-induced obesity (DIO) and may contribute to the reduced nuclear β-catenin levels observed in *db/db* mice. Transcriptomic profiling further revealed that obesity alone preferentially activates inflammatory and stress-response pathways while suppressing lipid-sensing nuclear receptor programs. These findings underscore a critical distinction between HFD exposure and obesity as biological variables and suggest that systemic metabolic dysfunction is insufficient to drive epithelial membrane remodeling in the absence of excess dietary lipid.

The rapid onset of membrane remodeling following just 1 week of HFD feeding further supports a diet-driven mechanism. Acute HFD exposure increased membrane rigidity in colonocytes and splenic CD4^+^ T cells prior to detectable changes in body weight or adiposity, indicating that membrane adaptation is an early and potentially reversible response to dietary lipid availability. Interestingly, this early phase was associated with reduced circulating HDL and LDL cholesterol and transcriptional suppression of cholesterol biosynthesis pathways, suggesting transient redistribution of lipid pools and early intestinal adaptations in lipid handling. These responses are distinct from the sterol accumulation and hyperproliferative reprogramming observed under chronic HFD conditions. Notably, increased membrane rigidity occurred in the absence of any change in free cholesterol abundance. This observation suggests that membrane biophysical properties may be altered before measurable changes in total cellular cholesterol become apparent. Potential explanations include redistribution of cholesterol within the plasma membrane, altered cholesterol accessibility, or nanoscale lipid reorganization below the detection threshold of Filipin III staining. Consistent with this interpretation, LDL cholesterol–treated YAMC cells exhibited significant increases in membrane rigidity at concentrations (1 μg/mL) that did not significantly alter Filipin III fluorescence (Figure 10*A* and *B*).

Our in vitro data show that cholesterol is sufficient to induce membrane rigidification in colonic epithelial cells. Exogenous LDL cholesterol treatment increased plasma membrane cholesterol and rigidity in YAMC cells, recapitulating the biophysical phenotype observed in vivo. These findings conceptually align with evidence that LDL exposure can enhance ISC stemness ex vivo and alter intestinal organoid behavior.^49^ In contrast, exposure to saturated (PA) or monounsaturated (POA) fatty acids failed to induce sustained membrane rigidification and instead modestly decreased membrane rigidity. These findings are consistent with emerging evidence that mammalian cells actively buffer membrane composition against perturbations from saturated and monounsaturated fatty acids,^22^ whereas unesterified (free) cholesterol availability remains a dominant determinant of membrane rigidity.^16^ Long-term PA treatment in human colonic organoids produced only transient changes in membrane cholesterol without altering rigidity, reinforcing the notion that fatty acids alone are insufficient to drive stable membrane remodeling.

Single-cell transcriptomic analyses further support a model in which HFD promotes a stem-like, hyperproliferative state within the ISC compartment. For example, HFD ISCs exhibited increased expression of mitotic regulators, Wnt pathway components, and growth factor–responsive genes, along with elevated cell potency scores. Importantly, UCell scoring revealed coordinated upregulation of sterol and cholesterol biosynthesis pathways coupled with suppression of cholesterol efflux and homeostatic response programs. Although transcriptional data do not directly measure lipid flux, the data are consistent with perturbed cholesterol handling that may favor intracellular and membrane cholesterol accumulation. These ISC-specific transcriptional signatures parallel upstream regulator patterns observed in bulk colonocyte RNA-seq, corroborating diet-induced regulatory signals within the ISC compartment. Additionally, because proliferation and membrane biogenesis are mechanistically coupled,^50^ the observed upregulation of cholesterol and mevalonate pathway activity provides a plausible link between altered sterol handling and the heightened proliferative demands of the HFD-induced ISC state. In this context, enhanced sterol biosynthesis may support both membrane expansion and sustained cell–cycle progression, reinforcing a chronic shift toward a proliferative, stem-like transcriptional program that is concordant with the membrane remodeling and enhanced Wnt and EGFR signaling phenotypes identified by imaging and functional assays. This coupling between cholesterol metabolism and proliferation also aligns with emerging concepts of membrane homeostasis, wherein sterol biosynthesis and membrane expansion are coordinated to meet the structural and signaling demands of dividing cells.^50^^,^^51^ Thus, HFD-induced cholesterol accumulation may simultaneously stiffen the plasma membrane, reorganize receptor nanodomains, and supply the lipid substrates required for sustained membrane biogenesis during stem cell expansion.

Altogether, our novel findings support a model in which HFD-associated lipid composition, likely driven in part by cholesterol present in animal-based fat sources, acts as a critical mediator of epithelial membrane remodeling, enhancing plasma membrane rigidity and reorganizing receptor nanodomains to potentiate Wnt and EGFR signaling. This membrane-centric mechanism provides a structural basis for how dietary lipid exposure can amplify stem cell signaling and proliferative capacity, potentially lowering the threshold for tumor-initiating events in the colonic epithelium. In support of this concept, recent work in *Drosophila*, a sterol auxotroph, demonstrates that dietary cholesterol directly enhances ISC proliferation, humanized LRP6 and Fzd receptor nanoclustering, and Wnt signaling reporter assay through altered membrane dynamics,^33^ highlighting an evolutionary conservation of cholesterol-sensitive epithelial regulation. In the same study and consistent with these findings, cholesterol supplementation of a Wnt reporter cell line (Leading Light 3T3 cells) induced a dose-dependent increase in Wnt/β-catenin reporter activity, whereas pharmacologic cholesterol depletion using mevastatin or MβCD attenuated Wnt signaling, indicating that membrane cholesterol availability directly tunes pathway responsiveness.^33^

Several limitations of this study merit consideration. First, although our combined imaging and biochemical approaches robustly capture membrane-level changes, we did not directly manipulate cholesterol handling in vivo using genetic, pharmacologic, or dietary approaches, which will be essential to establish causality. Second, mechanistic analyses were restricted to male mice because only males exhibited robust metabolic responses to HFD feeding under the conditions tested. Although both sexes were initially included, female mice failed to develop the robust metabolic response to HFD feeding, consistent with the known resistance of female C57BL6 mice to DIO. As a result, the present study cannot determine whether HFD-driven membrane remodeling and stem cell activation occur independently of sex or whether these effects are modulated by sex-specific hormonal or metabolic factors. Thus, future studies are required to determine whether sex-specific differences in lipid handling and membrane regulation influence intestinal stem cell responses to dietary fat in female mice. Third, scRNA-seq analyses were performed on pooled samples, with cells from 3 mice per diet group combined prior to library preparation. Consequently, these data are best interpreted as cell-state and transcriptional differences rather than interanimal variability or diet-associated changes in cell-type abundance, emphasizing transcriptional states rather than population dynamics. Finally, although we demonstrate strong parallels between membrane remodeling and stem cell activation, tumor initiation and progression were not directly assessed in this study and remain important areas for future investigation.

In summary, this work identifies plasma membrane remodeling as a rapid, diet-sensitive regulator of colonic stem cell behavior that operates independently of obesity. By linking HFD-associated lipid exposure to membrane biophysics, receptor nanoclustering, and stem cell signaling, our findings provide new insight into the earliest cellular events through which HFDs may increase colorectal cancer risk. Future studies examining dietary cholesterol manipulation in vivo, independent of total fat intake, are essential for defining its specific contribution to membrane remodeling and epithelial tumor susceptibility. These results highlight the plasma membrane as a previously underexplored therapeutic target for mitigating diet-associated epithelial dysregulation and cancer susceptibility.

## Materials and Methods

### Mice Genetics, Husbandry, and Diet

All experiments utilizing mice were approved and conducted in accordance with the Texas A&M University Institutional Animal Care and Use Committee and conformed to National Institutes of Health guidelines. C57BL6 wild-type (WT), Lgr5-EGFP-IRES-CreERT2 (Lgr5-GFP), and Lepr^db^ (*db/db*) mouse strains were utilized. Specifically, Lgr5-GFP mice express EGFP and Cre^ERT2^ in crypt base columnar stem cells in the intestine under control of the Lgr5 promoter.^52^ Multiple cohorts of C57BL6 WT and Lgr5-GFP mice were maintained on LFD (Research Diets, D12450B) and a subset were switched to a HFD (Research Diets, D12492) at the initiation of select experiments. Initial DIO experiments involving C57BL6 WT mice were purchased from Jackson Laboratories (stock number 380050) at 17 weeks of age (11 weeks on diet) and upon arrival were acclimated for 1 week prior to being euthanized at 18 weeks of age (12 weeks on diet). Leptin receptor knockout male mice (*db/db*) and their littermate controls (*db/+*) consuming a chow diet (LabDiets, 5K52) were purchased from Jackson Laboratories (stock number 000697) at 10 weeks of age and upon arrival were acclimated for 1 week on the LFD (Research Diets, D12450B) prior to being euthanized at 11 weeks of age. Mice were provided with fresh diet every week. All mice were fed ad libitum and housed in microisolator cages in a temperature- and humidity-controlled animal facility with a 12-hour light and dark cycle. Mouse body composition analysis was performed utilizing an EchoMRI-100H instrument. Mice were euthanized by either CO_2_ asphyxiation followed by cervical dislocation or by anesthetized cardiac exsanguination followed by cervical dislocation.

### Colonic Crypt and Single-Cell Colonocyte Isolation

Mouse colons were collected, rinsed with ice-cold Dulbecco’s Phosphate Buffered Saline (DPBS; Gibco, 14190144) without calcium and magnesium, everted on a disposable mouse gavage needle (Instech Laboratories, FTP-20-38), and incubated in 15 mM EDTA (Sigma, ED4SS) dissolved in DPBS, at 37 °C for 35 minutes as previously described.^53^ Subsequently, following transfer to chilled DPBS, crypts were mechanically separated from the connective tissue by rigorous vortexing. Crypt suspensions were dissociated into single cells utilizing 0.25% Trypsin-EDTA (Gibco, 25200056) or 10X TrypLE Select Enzyme (Gibco, A1217701). Cell suspensions were then filtered through a 40-μm cell strainer immediately prior to sorting. GFP^+^ cells were collected using a MoFlo Astrios Cell Sorter (Beckman Coulter) or S3e Cell Sorter (Bio-Rad). Dead cells were excluded by staining with propidium iodide (PI) or 7-aminoactinomycin D. Sorted cells were collected in Live Cell Imaging Solution (LCIS; Invitrogen, A14291DJ) as previously described.^54^

### Colon Tissue Fixation and Immunohistochemistry

Mouse colons were collected, rinsed with ice-cold DPBS, cut longitudinally with scissors, rolled on histologic paper into Swiss rolls, and placed in 4% paraformaldehyde (PFA) to be fixed on ice for 4 hours. Tissues were subsequently washed with ethanol prior to being embedded in paraffin. Embedded colon samples were sectioned onto Superfrost Plus microscope slides (Fisher, 12-550-15) and antigen retrieval was performed by sub-boiling in 10 mmol/L sodium citrate (pH 6.0) for 20 minutes. Antibodies used were goat polyclonal antibody to GFP (Abcam, ab6673) followed by Alexa Flour Plus 488 donkey anti-goat secondary antibody (Invitrogen, A32814), mouse monoclonal antibody to β-catenin (BD Biosciences, 610154) followed by CF640R conjugated secondary antibody against mouse (Biotium, 20178). ProLong Gold antifade mountant with 4′,6-diamidino-2-phenylindole (Invitrogen, P36931) was used to coverslip the slides. For nuclear β-catenin assessment, nuclei regions of interest were created utilizing the 4′,6-diamidino-2-phenylindole stain.

### Splenic T Cell Isolation and Enrichment

Mouse spleens were collected, and CD4^+^ and CD8^+^ T cells were isolated by positive selection using magnetic CD4 (L3T4) (Miltenyi Biotec, 130-049-201) or CD8 (TIL) (Miltenyi Biotec, 130-116-478) microbeads according to the manufacturer’s protocol. Isolated T cells were resuspended in LCIS and utilized for membrane rigidity and free cholesterol experiments.

### Blood Serum Analysis

Nonfasted mice were anesthetized with tribromoethanol, cardiac blood was collected, and mice were euthanized. Blood samples, collected into 2.0-mL tubes (USA Scientific, 1620-2799), were allowed to clot at room temperature (RT) for 30 minutes, then were centrifuged at 1500 × g for 15 minutes. The serum was transferred to a new tube and stored at −80 °C. Serum levels of total cholesterol, HDL cholesterol, LDL cholesterol, and triglycerides were measured using a Beckman-Coulter DxC 700 AU Chemistry Analyzer.

### Cell Culture

Conditionally immortalized YAMC epithelial cells were originally obtained from R.H. Whitehead, Ludwig Cancer Institute. YAMC cells (passages 12−20) were cultured under permissive conditions, 33 °C and 5% CO_2_ in RPMI 1640 medium without glutamine (Gibco, 21870076) supplemented with 5% fetal bovine serum (Hyclone, SH300084.03), 2 mM GlutaMAX (Gibco, 35050061), 5 μg/mL insulin, 5 μg/mL transferrin, 5 ng/mL selenious acid (Corning, 354351), and 5 IU/mL of murine interferon-γ (Roche, 11276905001). YAMC cells were authenticated by STR profiling (CellCheck Plus) by IDEXX BioResearch. All cell lines used tested negative for mycoplasma bacteria as assessed by a Universal Mycoplasma Detection Kit (ATCC, 30-1012K). Select cultures were serum starved for 24 hours prior to being treated for 24 hours with 5, 10, 20, or 50 μM of human LDL cholesterol (Kalen Biomedical, 770200-1). Select cultures were treated for 72 hours with 50 or 250 μM of PA or POA complexed to BSA, prepared as previously described.^55^ Select organoid cultures were treated with 30 μM of PA complexed to BSA for up to 29 days. In addition, select cultures were incubated with complete media containing 10 mM MβCD (Sigma, C4555) for 30 minutes to deplete cholesterol from the plasma membrane.

### Organoid Culture

Sorted Lgr5-GFP^high^ and Lgr5-GFP^low^ cells were embedded in Matrigel and overlaid with crypt culture media as previously described.^53^ Organoid culture medium was composed of Advanced Dulbecco’s Modified Eagle Medium–F12 (Gibco, 12634010) supplemented with 2 mM GlutaMAX, 10 mM HEPES (Sigma, H0887), 50 ng/mL EGF (Life Technologies), 0.2 μM LDN-193189 (Stemgent, 04-0074), 10% R-spondin-1 conditioned medium, 1 μM N-acetyl-L-cysteine (Sigma, A9165), 1X N2 (Life Technologies, 17502048), 1X B27 (Life Technologies, 17504044), and 50% Wnt conditioned medium as described previously.^56^ Sorted intestinal cells were centrifuged for 3 minutes at 500 × g, resuspended in the appropriate volume of Matrigel (20-30 μL) containing 10 μM Y-27632 (Sigma, Y0503) and 1 μM Jagged-1 (AnaSpec, AS-61298) in flat bottom 24-well or 48-well plates. Following Matrigel polymerization, cells were overlaid with 500 μL of crypt culture medium supplemented with 10 μM Y-27632, 1 μM Jagged-1, and 2.5 μM CHIR99021. Y-27632, Jagged-1 and CHIR99021 were withdrawn from culture medium 2 days after plating. The crypt media was changed every 2 days.

For human organoid cultures, Advanced Dulbecco’s Modified Eagle Medium–F12 was supplemented with 2 mM GlutaMAX, 10 mM HEPES, 40 ng/mL EGF (Life Technologies, PMG8041), 3 μM SB202190 (Sigma, S7067), 500 nM A83-01 (Tocris, 2939), 10 mM nicotinamide (Sigma, N3376), 1 μM N-acetyl-l-cysteine, 1X N2, 1X B27, 10 nM gastrin I (Sigma, G9020), 100 μg/mL Primocin (Invitrogen, ant-pm), and 50% Wnt-3a, R-spondin 3, and Noggin (WRN) conditioned medium as previously described.^54^ Following Matrigel polymerization, cells were overlaid with culture media supplemented with 10 μM Y-27632, 1 μM Jagged-1, and 2.5 μM CHIR99021. Y-27632, Jagged-1 and CHIR99021 were withdrawn from culture medium 2 days after plating. The crypt media was changed every 2 days.

### Membrane Order Measurement via Confocal Microscopy

Colonic crypts were stained with 5 μM Di4 (Invitrogen, D36802) for membrane order and rigidity determination as previously described.^57^, ^58^, ^59^, ^60^, ^61^, ^62^, ^63^ In brief, Di4 was added to colonic crypts in LCIS. Stained crypts were transferred into an 8-chamber cell imaging cover glass (Eppendorf, 0030742036; Cellvis, C8-1.5H-N) and imaged immediately to avoid dye internalization. Imaging experiments were conducted using a Leica DMi8 confocal microscope. Colonic crypts were imaged with a 0.6 numerical aperture (NA) 20× Plan Apochromat oil objective at RT. Laser light at 488 nm was used to excite Di4 and emission wavelengths were collected in 2 channels representing ordered (O: 500-580 nm) and disordered (D: 620-700 nm). GP was calculated using the equation below:

GP=(Intensity(O)−G×Intensity(D))/(Intensity(O)+G×Intensity(D))

Here, G is a calibration factor determined using a solution of 500 μM Di4 in dimethyl sulfoxide following a procedure described previously.^62^ A reference value of −0.85 was used for Di4. The same laser power and detector settings were used for every experiment. Image processing was conducted using Fiji and ImageJ (NIH) software, with a GP-plugin and a custom-built macro. Briefly, images were converted to 8-bit tiffs, combined into RGB images. A threshold was applied to exclude background pixels and images were converted into GP images. Average GP values were determined from regions of interest containing a single colonic crypt to exclude cellular debris.

### Membrane Order Measurement via Image-Based Flow Cytometry

Single-cell colonocytes and T cells were stained with 1 μM Di4 for membrane order and rigidity determination as previously described.^57^, ^58^, ^59^, ^60^, ^61^, ^62^, ^63^ In brief, Di4 was added to single-cell colonocytes and T cells in LCIS. Stained cells were imaged immediately to avoid dye internalization. Imaging experiments were conducted via image-based flow cytometry, Amnis FlowSight (Cytek Biosciences)^55^^,^^59^^,^^63^ containing a 0.6 NA 20× objective. Laser light at 488 nm was used to excite Di4, and emission wavelengths were collected in 2 preset channels representing order (O: 480–560 nm) and disorder (D: 640–745 nm). Because there is no way to acquire a calibration image, the G factor was omitted, and GP was calculated using the equation below via Amnis IDEAS 6.2 software.

GP=(Intensity(O)−Intensity(D))/(Intensity(O)+Intensity(D))

### Filipin III Unesterified Cholesterol Staining

Colonic crypts, single-cell colonocytes, and T cells were fixed in PFA (Electron Microscopy Sciences, 15713-S) diluted to 4% in PEM buffer (80 mM PIPES, 5 mM EGTA, 2 mM MgCl_2_, pH 6.8) for 20 min on ice. Subsequently, cells were washed with DPBS and 50 μg/mL Filipin III (Sigma, SAE0087) was added for 45 minutes at RT in the dark as previously described.^64^ After 45 minutes, excess Filipin III was removed by washing with DPBS. Colonic crypts were imaged with a 0.6 NA 20× Plan Apochromat oil objective on a wide-field Nikon Eclipse microscope, with a 0.45 NA 20× Plan Fluorite air objective on a Keyence BZ-X710 all-in-one fluorescence microscope, or with a 0.6 NA 20× objective on a FlowSight instrument. Quantitative image analysis was conducted using BZ-X Analyzer Hybrid Cell Count software (Keyence), Fiji and ImageJ (NIH) software, or Amnis IDEAS software.

### Colonic Crypt Cholesterol Quantification

Lipids were extracted from ∼4000 isolated colonic crypts using a modified Folch extraction procedure.^63^ Following phase separation, the organic fraction containing lipids was collected, dried under nitrogen, and resuspended in assay buffer for cholesterol quantification. Free cholesterol and total cholesterol were measured using the Amplex Red Cholesterol Assay Kit (Thermo Fisher Scientific, A12216) according to the manufacturer's instructions. For free cholesterol measurements, lipid extracts were incubated with Amplex Red reagent, horseradish peroxidase, cholesterol oxidase, and cholesterol assay buffer. Total cholesterol measurements were performed in parallel reactions containing cholesterol esterase to hydrolyze cholesteryl esters prior to cholesterol oxidation. Fluorescence was measured using a CLARIOstar microplate reader (BMG LABTECH), and cholesterol concentrations were determined from cholesterol standard curves generated in parallel. Cholesteryl ester abundance was calculated as the difference between total cholesterol and free cholesterol measurements. To normalize for lipid input, lipid phosphate content was quantified from the same lipid extracts using the Phosphate Assay Colorimetric Kit (Abcam, ab65622) according to the manufacturer’s instructions. Briefly, lipid samples were subjected to acid hydrolysis to liberate inorganic phosphate from phospholipids, followed by colorimetric detection of phosphate. Phosphate concentrations were calculated from phosphate standard curves generated in parallel. Free cholesterol, total cholesterol, and cholesteryl ester values were normalized to phospholipid phosphate content and presented as cholesterol-to-phosphate ratios. This normalization accounts for differences in lipid recovery and sample loading between preparations and provides a measure of cholesterol abundance relative to total membrane phospholipid content.

### High-Performance Liquid Chromatography–Tandem Mass Spectrometry Lipidomics

Whole cell pellets (∼4000 colonic crypts) from 5 HFD-fed and 5 LFD-fed male mice were utilized to extract lipids. Samples were spiked with EquiSPLASH LIPIDOMIX Quantitative Mass Spec Internal Standards (Avanti, #330731-1EA) prior to Folch lipid extraction. A small volume, 35–40 μL, of Folch solution containing the lipid samples and internal standards was filtered utilizing a syringe attached to a syringe filter (0.22 μm pore size, 25 mm diameter, hydrophilic polytetrafluoroethylene membrane). At least 20 μL of solution was prepared and filtered for analysis. Mass spectrometric analysis was performed on Orbitrap Velos Pro (ThermoFisher Scientific). A vanquish UHPLC system (ThermoFisher Scientific) coupled with a Orbitrap Velos Pro mass spectrometer (ThermoFisher Scientific) was applied for LC-MS analysis of all samples. An aliquot of 5 μL of sample was injected onto the Accucore C30 column (ThermoFisher Scientific, C30, 2.1 mm × 150 mm, 2.6 μm). The mobile phase was acetonitrile and water (60:40, v:v) (solvent A) and isopropanol and acetonitrile (90:10, v:v) (solvent B), both containing 10 mM ammonium acetate and 0.1% formic acid. The column separation was carried out at 40 °C with a flow rate of 0.4 mL/min. The elute gradient was: 30% B at 0–3 minutes, 30%–43% B at 3–5 minutes, 43%–55% B at 5–5.1 minutes, 55%–65% B at 5.1–15 minutes, 65%–85% B at 15.1–21.0 minutes, 85%–100% B at 21.1–23 minutes, 100% B at 23.1–28 minutes, 100%–30% B at 28.1–30.0 minutes, and 30% B at 30–35 minutes. Sheath gas, auxiliary gas and sweep gas flow rate were set as 40, 7, and 1 arb, respectively. The applied electrospray voltage was 3 kV, and the temperature of the ion transfer capillary was 300 °C, whereas the S-lens RF Level was 60%.

HPLC-MS/MS data were processed utilizing the Thermo Xcalibur Qual Browser Program and Thermo LipidSearch. Adducts from positive and negative ion modes were conflated and aligned, and only lipid species found in the internal standard and in common in all samples were taken into consideration (PC, LPC, PE, LPE, PG, PI, PS, TAG, DAG, SM, and Cer). Furthermore, only lipid species found in the plasma membrane were utilized for downstream analyses (PC, LPC, PE, LPE, PG, PI, PS, SM, and Cer). Lipidomic data were expressed as mole percent (mol%). The unsaturation index was calculated as a concentration-weighted average of acyl-chain double bonds across membrane phospholipid species, determined by multiplying the relative abundance of each lipid by its total number of double bonds and summing across species.^22^

### 5-Ethynyl-2′-Deoxyuridine Administration and Staining

To assess cell proliferation in the colon, mice were intraperitoneally injected with EdU 2 hours prior to being euthanized as previously described.^65^ Colonic cell proliferation was measured using a Click-iT EdU kit (Life Technologies, C10340).

### Super Resolution Stochastic Optical Reconstruction Microscopy

Mouse primary single colonocytes were suspended in LCIS and seeded onto poly-D-lysine (Gibco, A3890401) coated 8-well coverglass slides (Cellvis, C8-1.5H-N) at a concentration of 250,000 cells/well and allowed to attach for 30 minutes on ice. Following attachment, cells were fixed with ice-cold 4% PFA-PEM buffer for 15 minutes on ice. Following fixation, cells were washed with ice-cold DPBS twice. To fluorescently label colonocytes for nanocluster analysis using STORM, colonocyte cells were blocked with 5% BSA-DPBS for 30 minutes at RT and, incubated with 10 μg/mL of rat IgG2B LRP6 (R&D Systems, MAB2960) primary antibody in 1% BSA-DPBS for 1 hour at RT. Cells were rinsed with 1% BSA-DPBS twice, then rinsed with DPBS twice, blocked with 5% donkey serum-DPBS for 30 minutes at RT and incubated with 5 μg/mL donkey anti-rat CF568 secondary antibody in 1% donkey serum-DPBS for 1 hour at RT. Following incubation with secondary antibody, colonocytes were washed with 1% donkey serum-DPBS twice, then rinsed with DPBS twice. Alternatively, for the detection of EGFR, fixed colonocytes were incubated with 0.5 μg/mL of an Alexa Fluor 647 fluorescently labeled with murine EGF ligand in 1% BSA-DPBS for 1 hour at RT in the dark. Cells were rinsed with 1% BSA-DPBS twice, then rinsed with DPBS twice prior to imaging. Lastly, stained cells were fixed with 4% PFA-PEM for 15 minutes at RT and washed with DPBS twice. Samples were prepared for STORM utilizing a cysteamine (MEA; Sigma, 30070) glucose oxidase (Sigma, G2133) buffer described previously.^66^ Briefly, TN buffer (50 mM Tris at pH 8.0 and 10 mM NaCl), an oxygen scavenging system (0.5 mg/mL glucose oxidase, 40 μg/mL catalase [Sigma, C40], and 10% [w:v] glucose) and 10 mM MEA. MEA was stored as a solid at 4 °C and prepared fresh as a 1-M stock solution in 0.25 N HCl. This stock solution was kept at 4 °C and used within 2 weeks of preparation.

STORM imaging and analysis was performed using a Nikon N-STORM 4.0 Super-Resolution System. Samples were imaged at RT by direct STORM on an inverted NSTORM 4.0 Super-Resolution System (Nikon Ti) equipped with a 1.49 NA 100× Apochromat TIRF oil immersion objective and a quad-band pass dichroic filter (Nikon, 97335). Images were then acquired in highly inclined and laminated optical sheet microscopy mode, and focal drift was prevented with hardware autofocusing (Nikon Perfect Focus System 4). Alexa Fluor 647 was pushed into the dark state using the 640 nm (125 mW) laser line at maximum laser intensity. CF568 was pushed into the dark state using the 561 nm (125 mW) laser line at maximum laser intensity. Drift correction was performed using Nikon software. Data were acquired with a field-of-view 256 × 256 pixels (160 nm pixel size) at 8.5-ms frame rate for 10,000 frames with an EMCCD camera (Princeton Instruments, ProEM-HS 512BX3). The sparsity of single molecules per frame was controlled with 405 nm laser (100 mW) at 205–60% of total power. Single molecule localization analysis was performed using Nikon N-STORM software. The maximum width, which is the maximum possible width a spot of some intensity in the image to be identified as a molecule by STORM Analysis was set to 400 nm. To exclude the identification of 2 molecules in close proximity, fluorescent spots whose ratio of elongation in the X and Y direction was larger than 1.3 were rejected as a single molecule. Lateral drift was compensated for using autocorrelation-based drift correction with NIS-Elements AR software (v5.21.02), which corrects drift based on tracking the entire set of molecules. For nanocluster measurements, nanoclusters were identified by Voronoi analysis utilizing the NIS-Elements AR Analysis software (v5.21.02) which is based on 3D Delaunay triangulation.^67^ Briefly, Voronoi creates clusters from the molecules present in the current image with the specified maximum distance between the adjacent molecules and the specified minimum number of molecules in the emerging cluster. The maximum distance was set at 50 nm, and minimum number of molecules at 10 for both LRP6 and EGFR analysis. A masked image of the clusters was exported to ImageJ (v1.53f51), where it was converted to a binary image, and the analyze particle function was used to quantify clusters larger than 0.001 μm^2^.

### RNA Isolation, RNA Sequencing, and Ingenuity Pathway Analysis

Colonic crypts were resuspended in Buffer RLT RNeasy Lysis Buffer (Qiagen, 79216). RNA was extracted using RNeasy Micro Kit (Qiagen, 74004) and further processed using a DNA-free DNA removal kit (Invitrogen, AM1906) in accordance with the manufacturer’s protocol. RNA quality and integrity was assessed using a Bioanalyzer 2100 (Agilent Technologies), quantified by Nanodrop, and stored at −80 °C. Only RNA quality with an RNA Integrity Number ≥8 was used. Samples were subsequently sequenced using TruSeq Illumina Stranded mRNA Kit (Illumina). Sequencing was performed on a NextSeq 500 (Illumina). Following the removal of all genes with counts-per-million reads values less than 1, publicly available R software (v4.1.2), EdgeR robust (v3.34.1), was used to identify DEGs.^68^ To determine differentially regulated pathways and predict upstream regulators, IPA (Qiagen, v01-23-01) was utilized. DEGs were identified using an absolute log2fold change (FC) ≥0.5, *P* ≤ .05, and adjusted *P* (false discovery rate [FDR]) ≤ .05 when compared with their respective control.

### Single-Cell RNA Sequencing Sample Preparation

Single-cell suspensions were gently pipetted 10–15 times through a wide-bore pipet tip to break up clumps, followed by filtering through a 30-μm CellTrics filter (Sysmex America) into 1.5-mL non-stick tubes (USA Scientific, 1415-2608). Mice (n = 3) were equally pooled into 1 sample per diet treatment. Samples were processed using the 10X Genomics scRNA-seq pipeline described below.

### 10X Genomics Single-Cell RNA Sequencing

Single-cell sample preparation was conducted according to Sample Preparation Protocol provided by 10X Genomics as follows: a cell suspension (1 mL) from each mouse genotype was pelleted by centrifugation (300 × g, 5 minutes). The supernatant was discarded, and the cell pellets resuspended in 1× phosphate-buffered saline with 0.04% BSA, followed by 2 washing procedures by centrifugation (300 × g, 5 minutes). Cells were resuspended in 500 μL 1× phosphate-buffered saline with 0.04% BSA followed by gently pipetting 10–15 times and enumerated using an Invitrogen Countess automated cell counter (Thermo Fisher Scientific), and the viability of cells was assessed by trypan blue staining (0.4%). Subsequently, single-cell gel beads in emulsion and sequencing libraries were prepared using the 10X Genomics Chromium Controller in conjunction with the single-cell 3′ kit (v3). Cell suspensions were diluted in nuclease-free water to achieve a targeted cell count of 10,000 for each sample. Complementary DNA synthesis, barcoding, and library preparation were carried out according to the manufacturer’s instructions. Libraries were sequenced by the North Texas Genome Center facility using a NovaSeq6000 sequencer (Illumina). For the mapping of reads to transcripts and cells, sample demultiplexing, barcode processing, and unique molecular identifier counts were performed using the 10X Genomics pipeline CellRanger v.8.0.0 with default parameters and the inclusion of introns. Sequences were aligned to the mouse reference genome (GRCm39-2024-A) to generate the gene-barcode matrix.

### Single-Cell RNA Sequencing Data Analysis

A gene-by-cell matrix was generated from the filtered feature gene barcode matrix utilizing the scGEAToolbox (v25.9.6) from GitHub: (https://github.com/jamesjcai/scGEAToolbox).^69^ Sample matrices were then merged before filtering out low-quality cells, cells expressing: (1) less than 1000 unique molecular identifiers or transcripts; (2) greater than 25% of mitochondrial reads; and (3) less than 500 unique genes were excluded utilizing the quality control filtering feature of the scGEAToolbox. Doublets (Scrublet v0.2.3)^70^ and ambient RNA contamination (decontX v1.2.0)^71^ were removed for quality control. Dimension reduction (*npcs* = 50) of expression matrices and cell clustering was performed using a 2D Uniform Manifold Approximation and Projection (*dims* = 30) and with Leiden clustering (*res* = 4.0) algorithms, respectively, utilizing the Seurat wrapper function in the scGEAToolbox. Batch correction was performed utilizing Harmony (v1.2.3).^72^ Cell type assignment was performed manually using common marker genes.^73^^,^^74^ Gene expression data were normalized by library size, which scales single-cell gene expression counts, by dividing each gene count by the total expression of its respective cell and multiplying by the mean library size across all cells. This ensured comparability across samples while accounting for differences in sequencing depth. Library size normalization was then followed by a log1p transformation prior to downstream analyses, unless stated otherwise. To identify DEGs between HFD and LFD colonocytes, we utilized the Differential Expression (DE) Analysis function in scGEAToolbox, which is based on the Wilcoxon rank sum (Mann–Whitney *U*) test and estimates the positive FDR by the Benjamini and Hochberg method in MATLAB R2024b (v24.2). Further, to examine the potential function of DEGs, we performed pathway enrichment analysis (Enrichr^75^) utilizing genes with a *P* adjusted value ≤ .05, and an absolute log2FC ≥1. To determine differentially regulated pathways and predict upstream regulators, IPA was utilized. DEGs were identified using an absolute log2FC ≥ 0.5, *P* ≤ .05, and adjusted *P* (FDR) ≤ .05 when compared with their respective control. The UCell package in R was used for evaluating the gene signature enrichment scores for a predefined gene set in single-cell datasets.^42^ The Molecular Signatures Database (MSigDB) v2023.2 was used as the source of annotated gene sets for analysis. Briefly, the UCell package allows the estimation of the gene signature enrichment scores called UCell scores (Mann–Whitney *U* statistic) for a given predefined gene set based on the expression of messenger RNA in a respective cell utilizing raw gene counts.

### Statistics

Unpaired, 1- or 2-tailed Student *t* tests, Mann–Whitney tests, and Holm–Sidak or Tukey multiple comparisons test were used to assess statistical significance of the differences between means across experimental treatments unless stated otherwise. All data are presented as mean ± standard error of the mean, unless stated otherwise, and all analyses were conducted using Prism 10 statistical software (GraphPad Software, Inc).

## Footnotes

Note: To access the supplementary material accompanying this article, visit the full text version at https://doi.org/10.1016/j.jcmgh.2026.101878.
